# Activating NEDD4L suppresses EGFR-driven lung adenocarcinoma growth via facilitating EGFR proteasomal degradation

**DOI:** 10.1186/s13046-025-03528-y

**Published:** 2025-10-21

**Authors:** Maojian Chen, Wei Jiang, Jianhua Zhan, Shaoping Zhang, Jiani Zheng, Yihua Huang, Junyi He, Yunpeng Yang, Shen Zhao, Yaxiong Zhang, Jiaqing Liu, Lanlan Pang, Li Zhang, Wenfeng Fang, Jing Li

**Affiliations:** 1 Department of Medical Oncology, State Key Laboratory of Oncology in South China, Guangdong Provincial Clinical Research Center for Cancer, Sun Yat-Sen University Cancer Center, Sun Yat-Sen university, Guangzhou, 510060 China; 2 Guangdong Provincial Key Laboratory of Malignant Tumor Epigenetics and Gene Regulation, Guangdong-Hong Kong Joint Laboratory for RNA Medicine, Sun Yat-Sen Memorial Hospital, Sun Yat-Sen University, Guangzhou, 510120 China; 3 Department of Respiratory Oncology, Guangxi Medical University Cancer Hospital, Nanning, 530021 China; 4 Department of Thoracic Surgery and Oncology, the First Affiliated Hospital of Guangzhou Medical University, State Key Laboratory of Respiratory Disease & National Clinical Research Center for Respiratory Disease, Guangzhou, 510120 China; 5 Department of Medical Oncology, School of Medicine, Xiamen Key Laboratory of Antitumor Drug Transformation Research, the First Affiliated Hospital of Xiamen University, Xiamen University, Xiamen, 361000 China

**Keywords:** EGFR, NEDD4L, FOXM1, Osimertinib, Verteporfin, LUAD

## Abstract

**Background:**

Resistant mutations and amplification of the epidermal growth factor receptor (EGFR), followed by the upregulation of its translated protein undermines the efficacy of EGFR-tyrosine kinase inhibitors (TKIs) in EGFR-mutant lung adenocarcinoma (LUAD). This underscores that promoting EGFR protein degradation may be a promising strategy for treatment.

**Methods:**

Ubiquitin ligases database analysis identified NEDD4L as a mediator of EGFR proteasomal degradation, which was further confirmed by qPCR, western blot, immunofluorescence staining and CO-IP. The upstream regulatory role of FOXM1 on NEDD4L was elucidated through bioinformatics analyses and validated using dual luciferase reporter assay, ChIP, qPCR, western blot and immunohistochemistry. Virtual screening and molecular docking were used to identify inhibitors of FOXM1. Functional studies and therapeutic strategies were conducted using gain- and loss-of-function assays, and evaluated through in vitro and in vivo experiments.

**Results:**

We identified the E3 ubiquitin ligase NEDD4L that targets both wild-type EGFR and osimertinib-sensitive/resistant EGFR mutants for proteasomal degradation, thereby effectively inhibiting EGFR-driven LUAD growth. We found FOXM1 as a critical upstream transcription factor that binds to the promoter of NEDD4L and represses its expression, further promoting tumor growth and osimertinib resistance in LUAD by increasing EGFR protein level. High FOXM1 expression correlates with low NEDD4L expression in LUAD patients, which is associated with poor clinical outcomes. Notably, we further identified that verteporfin, an FDA-approved small molecule drug, as a FOXM1 inhibitor. Verteporfin suppresses FOXM1 to upregulate NEDD4L expression and facilitate EGFR proteasomal degradation, thereby inhibiting EGFR-driven LUAD growth and overcoming osimertinib resistance. Remarkably, the combination of verteporfin and osimertinib shows an additively inhibitory effect on EGFR-mutated LUAD growth compared to monotherapy, both in post-TKI resistance and upfront treatment settings.

**Conclusions:**

This study demonstrates that FOXM1/NEDD4L axis impairs EGFR proteasomal degradation, thus contributing to EGFR-driven LUAD growth and osimertinib resistance. Combination therapy incorporating NEDD4L activation may represent a new valued therapeutic strategy for EGFR-driven LUAD and osimertinib resistance.

**Supplementary Information:**

The online version contains supplementary material available at 10.1186/s13046-025-03528-y.

## Background

Lung cancer is the most deadly malignancy worldwide [1]. Lung adenocarcinoma (LUAD) is the predominant subtype of lung cancer [2]. EGFR mutations, particularly exon 19 deletions and L858R, are the major drivers in LUAD patients [3]. Despite the remarkable therapeutic benefits observed in LUAD patients harboring EGFR actionable mutations with the clinical application of first- and second-generation EGFR tyrosine kinase inhibitors (EGFR-TKIs), resistance ultimately develops due to the emergence of EGFR-T790M mutation in 50%-60% of patients [4]. Osimertinib, a third-generation EGFR-TKI, shows remarkable efficacy in treating patients with both EGFR actionable mutations and resistant mutations to earlier-generation EGFR-TKIs, particularly T790M [5, 6]. However, the emergence of resistance to osimertinib remains a significant challenge [7].

The most frequent on-target mechanisms of resistance to osimertinib have been reported to primarily involve acquired mutations within the EGFR tyrosine kinase domain and EGFR amplification [8]. Acquired point mutations in the C797 residue interfere with the binding and compromise the inhibitory activity of osimertinib [9]. Besides, other rare EGFR mutations, including G796X, L792X, L718X and G724S, also confer resistance to osimertinib [10]. Additionally, EGFR amplification has also been revealed following treatment with third-generation EGFR-TKIs, potentially contributing to the resistance to osimertinib [11]. Nevertheless, neither of these resistance mechanisms can be effectively addressed at the present. Consequently, there is an urgent need to further investigate the mechanism underlying EGFR-dependent TKI resistance, while concurrently exploring refined therapeutic strategies that have the potential to delay resistance and possibly reverse it.

The development of EGFR-TKIs, ranging from the first to the third, and even the fourth generation, has primarily targeted the ATP-binding site of EGFR. Unfortunately, the continuous emergence of resistant-mutations has ultimately led to disease progression. Recently, several studies have shown that stabilized EGFR accelerates tumor initiation and progression of EGFR-driven NSCLC, while destabilizing EGFR protein generates the inhibitory effect [12, 13], indicating that promoting EGFR protein degradation may be a potential alternative therapy to target EGFR-driven LUAD and overcome TKI resistance. Therefore, it is of profound importance to elucidate the mechanisms regulating EGFR protein degradation in LUAD. Previous studies have demonstrated that, in response to EGF stimulation, E3 ubiquitin ligase c-Cbl can induce EGFR mono-ubiquitination, endocytosis, and lysosome-mediated degradation, leading to the attenuation of EGFR signaling [14, 15]. Conversely, recent studies have reported that, in absence of EGF, proteasome-mediated degradation plays a crucial role in the regulation of EGFR protein stability [16, 17]. Nevertheless, the precise mechanisms underlying proteasome-dependent degradation of EGFR in LUAD remains to be fully elucidated.

Neural precursor cell-expressed developmentally down-regulated 4 like (NEDD4L), an E3 ubiquitin ligase belonging to the NEDD4 family [18, 19], has been reported to be downregulated in various cancers and clinically correlated with cancer prognosis [19–22]. Recently, increasing number of studies have highlighted the role of NEDD4L in regulating tumor initiation and progression by modulating the stability of its substrate proteins [23–25]. However, whether NEDD4L is involved in regulating the stability of the membrane oncoprotein EGFR and contributes to the development LUAD remains largely unknown.

FOXM1 (Forkhead box protein M1), a pivotal proliferation-associated transcription factor, has been observed to be upregulated in a variety of human cancers [26–29]. FOXM1 has been reported as a crucial regulator of cell cycle progression, and several studies have established its pivotal role in promoting LUAD growth [29–31]. Recently, accumulating studies have revealed that FOXM1 also exerts a significant impact on the resistance to chemotherapy [32], targeted therapy [33] and radiotherapy [34] in cancer cells. Noteworthily, our recent study identified a genetic variant, rs3742076_G, within FOXM1 that influences the variability in patient responses to gefitinib therapy and highlighted this variant as a promising predictive biomarker for gefitinib resistance in NSCLC [35].

In this study, we discovered that EGFR amplification may contribute to the resistance to the third-generation EGFR-TKIs in EGFR-mutated LUAD. Furthermore, our findings revealed that FOXM1/NEDD4L axis impairs EGFR proteasomal degradation, thereby contributing to EGFR-driven LUAD growth and osimertinib resistance. Notably, we further identified verteporfin as a FOXM1 inhibitor, suppresses FOXM1 to upregulate NEDD4L expression and facilitates EGFR proteasomal degradation, thereby inhibiting EGFR-driven LUAD growth and overcoming osimertinib resistance. Consequently, combination therapy incorporating NEDD4L activation may represent a new valued therapeutic strategy for EGFR-driven LUAD and osimertinib resistance.

## Methods

### Clinical samples

The study was approved by the Human Research Ethics Committees of Sun Yat-sen University Cancer Center. All lung cancer samples were collected with informed consent, and confirmed as LUAD by the Department of Pathology. EGFR mutation status was identified by polymerase chain reaction assays or next-generation sequencing (NGS). A substantial cohort that enrolled 153 EGFR-mutated LUAD patients between March 2019 and March 2023 was used to evaluate the effect of EGFR amplification on the efficacy of the third-generation EGFR-TKIs as the first-line treatment. Eligible patients were aged ≥ 18 years; pathologically diagnosed stage IIIB or IV LUAD harboring sensitive EGFR mutations (L858R and 19del); received the third-generation EGFR-TKIs as the first-line treatment (including osimertinib, aumolertinib and furmonertinib). Exclusion criteria included, previous treatment before the third-generation EGFR-TKI; in combination with other anti-tumor agents (e.g. cytotoxic agents, or anti-angiogenesis inhibitors); clinically significant cardiovascular and cerebrovascular diseases, or active infections; incomplete radiographic or follow-up data. Response assessments were performed by using computed tomography or magnetic resonance imaging according to Response Evaluation Criteria in Solid Tumors v.1.1 (RECIST v.1.1) [36].

In addition, for western blot analysis, another cohort of twelve LUAD samples with the adjacent normal tissues were taken at the time of surgery and quickly frozen in liquid nitrogen and stored at − 80 °C. For immunohistochemical (IHC) analysis, commercially available tissue microarrays consisting of forty-eight LUAD tissues and matched adjacent tissues used in this study were purchased from Shanghai Weiao Biotechnology Co., Ltd (Shanghai, China; ZL-LUgA961).

### Bioinformatics analysis

Gene expression data and corresponding clinical information from LUAD patients were downloaded from the Cancer Genome Atlas (TCGA) database (https://cancergenome.nih.gov). The gene expression profile of GSE31210 dataset was downloaded from Gene Expression Omnibus (GEO) database (https://www.ncbi.nlm.nih.gov/gds). UbiBrowser 2.0 [37] (http://ubibrowser.bio-it.cn/ubibrowser_v3/) online database was used to predict the potential E3 ubiquitin ligases that degrades EGFR protein. The Kaplan–Meier Plotter [38] (http://kmplot.com) online database was utilized to evaluate the overall survival (OS) probability between high and low expression groups of NEDD4L or FOXM1 in LUAD. MotifMap [39] (https://motifmap.ics.uci.edu/) online database was applied to mapping the position weight matrix (PWM) for FOXM1 Binding motif sequence. KnockTF 2.0 [40] (http://www.licpathway.net/KnockTF/index.html) and hTFtarget [41] (http://bioinfo.life.hust.edu.cn/hTFtarget) online database were used to predict potential upstream transcription factors of NEDD4L, and hTFtarget online database was utilized to predict FOXM1-binding site in the promoter region of NEDD4L.

### Antibodies and reagents

Antibodies against the following proteins were used for western blot: NEDD4L (Cat. #13690–1-AP, Proteintech; 1:2000), FOXM1 (Cat. #13147–1-AP, Proteintech; 1:3000), EGFR (Cat. #4267, Cell Signaling Technology; 1:1000), phosphorylated EGFR (p-EGFR) (Cat. #3777, Cell Signaling Technology; 1:1000), GAPDH (Cell Signaling Technology; Cat. #5174, 1:1000), Flag-tag (Cat. #14793/8146, Cell Signaling Technology; 1:1000), HA-tag (Cat. #2367/3724, Cell Signaling Technology; 1:1000), His-tag (Cat. #12698, Cell Signaling Technology; 1:1000), Ki67 (Cat. #ab16667,Abcam; 1:500), LC3B (Cat. #83506, Cell Signaling Technology; 1:1000). HRP-conjugated rabbit/mouse IgG (Cat. #W4011/W4021, Promega; 1:3000). Main reagents used in this study as follows: CHX (CAS: 66–81-9; Cat. #S7418), MG132 (CAS: 1211877–36-9; Cat. #S2619), Cycloheximide (CAS: 66–81-9; Cat. #S7418), Chloroquine (CLQ)(CAS: 54-05-7;Cat.#S6999), Osimertinib (CAS: 1421373–65-0; Cat. #S7297), FDI-6 (CAS: 313380–27-7; Cat. #S9689), Verteporfin (CAS: 129497–78-5; Cat. #S1786), Digoxin (CAS: 20830–75-5; Cat. #S4290), Glipizide (CAS: 29094–61-9; Cat. #S1715), Lomitapide (CAS: 182431–12-5; Cat. #S7635) were purchased from Selleck Chemicals. Ketotifen (CAS: 34580–13-7; Cat. # K922344) were purchased from Macklin Inc. Recombinant Human EGF (Cat. #C029) were purchased from Novoprotein Scientific Inc.

### Structure-based virtual screening

To identify potential FOXM1 inhibitors, AutoDock Vina was used for structure based virtual screening. The X-ray crystal structure of human FOXM1 (PDB ID: 3G73) was obtained from RCSB Protein Data Bank and further processed by the Protein Preparation Wizard module of Discovery Studio (DS) 2019. An FDA approved drug Library containing 1300 small molecule structure files were downloaded from Selleck (https://www.selleck.cn/). After energy optimization was conducted, virtual screening based on molecular docking to screen the conformations of ligands with high affinity at the FOXM1 binding site was carried out through PyRx software (GUI version 0.8). Docking was performed by keeping the target protein rigid while the ligands were kept flexible. The ligands were shortlisted based on the protein–ligand complex with the lowest binding energy and drug class. Upon completion of the analyses, five candidate compounds were selected for experimental validation. PyMOL software was utilized to generate visualization of the resulting plots.

### Cell culture

Human lung adenocarcinoma cell lines (A549, H1299, PC9, HCC827, H1975), normal lung epithelial cell line (BEAS-2B) and Human embryo kidney (HeK) 293 T cell lines were obtained from American Type Culture Collection (ATCC, Manassas, VA, USA). Osimertinib-resistant LUAD cell line (H1975OR) (bearing EGFR-L858R/T790M/C797S mutation) was induced and stored in the laboratory and the emergence of the C797S mutation was validated by sanger sequencing (Supplementary Fig. S1A). All cell lines were tested for mycoplasma contamination using a commercially available PCR-based assay and were confirmed to be negative for mycoplasma. A549, H1299, PC9, HCC827, H1975, H1975OR and BEAS-2B cells were maintained in Roswell Park Memorial Institute (RPMI)-1640 medium (GIBCO, Carlsbad, CA). HEK293T cells were cultured in Dulbecco’s modified Eagle’s medium (DMEM) (GIBCO, Carlsbad, CA). Both media were supplemented with 10% fetal bovine serum (FBS; Gibco, Rockville, MD, USA), and 1% penicillin–streptomycin (Hyclone, Logan, UT, USA) and grown at 37 °C in a Humidified 5% CO_2_ atmosphere in an incubator.

### Plasmids, siRNAs, shRNAs and cell transfection

The CDS regions of human EGFR, NEDD4L or FOXM1 were synthesized using PCR amplification and inserted into a pcDNA3.1 or pReceiver-Lv105 (GeneCopoeia, Guangzhou, China) vector with or without a tag (HA, Flag, His). To generate constructs with specific mutations in EGFR (19del (E746-A750del), L858R, T790M, C797S, L718Q, 19del/T790M, 19del/T790M/C797S, L858R/T790M, L858R/T790M/C797S, ECD, ICD), and NEDD4L (C2^del^, WW^del^, HECT^del^, C924A), forward primers containing mutated sites were used during PCR amplification. All the constructs including mutants generated by QuickMutation™ Site-Directed Mutagenesis Kit (Cat. #D0206, Beyotime, China) were confirmed by sanger sequencing. The short hairpin RNAs (shRNAs) targeting human EGFR, NEDD4L, FOXM1 were generated by the insertion of specific oligos into a pLKO.1-puromycin or blasticidin lentiviral vector. The target sequences of si/shRNAs used in this study were listed in Supplementary Table S2. For lentiviruses production, corresponding lentiviral plasmids together with packing plasmids psPAX2 and pMD2.G were co-transfected into HEK293T cells. Viruses were collected at 48 h and 72 h after transfection. For transient transfection, cells were transfected with siRNAs or plasmids using Lipofectamine™ 3000 reagent (Invitrogen, MA, USA) according to the manufacturers’ instructions. The knockdown or overexpression efficiency was evaluated by real time PCR or western blot.

### Chromatin immunoprecipitation assay

The chromatin immunoprecipitation (ChIP) assay was performed using a BeyoChIP™ Enzymatic ChIP Assay Kit (Cat. #P2083S, Beyotime, China) according to the manufacturer's instructions. In brief, H1299 cells were lysed using SDS lysis buffer and DNA was sheared by sonication. Protein-DNA complexes were precipitated by control immunoglobulin G (Cat. #30000–0-AP, Proteintech, Wuhan, China) and anti-FOXM1 antibody (Cat. #13147–1-AP, Proteintech, Wuhan, China) respectively, followed by eluting the complex from the antibody. Real time PCR was carried out with primers specific for NEDD4L promoter and normalized to the input.

### Promoter reporters and dual luciferase reporter assay

The NEDD4L promoter (-2000, +100) was amplified and the fragment was cloned into the luciferase reporter plasmids pGL3-basic vector (Promega, USA), designated as pGL3-NEDD4L. Mutant construct pGL3-NEDD4L-Mut was generated by site-directed mutagenesis. For luciferase assay, H1299 cells were seeded into 24-well plates and cultured without antibiotics overnight and then transfected with luciferase vectors or/and specified plasmids. In the meantime, all cells were co-transfected with pRL-SV40 Renilla luciferase construct for normalization. After 36 h, the supernatant was removed and cells were washed with PBS, subjected to lysis, and their luciferase activities were quantified using the Dual-Luciferase Reporter Assay System (Cat. #E2920, Promega, Madison, WI, USA).

### Real‑time quantitative PCR assay (qPCR)

Total RNA was extracted from cells using a minibest Universal RNA Extraction Kit (Cat. #9767, TaKaRa), and cDNA was synthesized from 1 μg of RNA with the PrimeScript™ RT reagent Kit (Cat. #RR047A, TaKaRa) according to the manufacturer’s instructions. Relative RNA levels of candidate genes were determined by SYBR Green Master Mix (Cat. #RR820A, Takara) in Light Cycler 480 II Real-Time PCR Systems (Roche). GAPDH was used as an internal control. The PCR primers sequences used in this study are shown in Supplementary Table S3. Relative quantification was performed using the comparative 2^−ΔΔ^ Ct method.

### Immunofluorescence (IF) staining

Cells were plated in confocal dishes (NEST Biotechnology) and incubated to 50% confluency, then washed with PBS, fixed with 4% paraformaldehyde for 20 min, permeabilized with 0.2% Triton X-100 for 10 min, blocked with QuickBlock™ Blocking Buffer for Immunol Staining (Beyotime, Shanghai, China) for 1 h at room temperature, and incubated with desired primary antibodies against EGFR (Cat. #4267, Cell Signaling Technology; 1:100), NEDD4L (Cat. #13690–1-AP, Proteintech; 1:200), HA-tag (Cat. #2367/3724, Cell Signaling Technology; 1:100) at 4 °C overnight, followed by incubation of fluorochrome-conjugated secondary antibodies (Beyotime, Shanghai, China) for 1 h at room temperature under dark, finally labeled by using DAPI (Beyotime, Shanghai, China) for 5 min, and subjected to a confocal fluorescence microscope (ZEISS,Jena,Germa).

### Hematoxylin and Eosin (H&E) staining

Lung tissue samples of mice were fixed with 4% paraformaldehyde, dehydrated with ethanol, immersed in xylene, embedded in paraffin and cut into 4.0 µm longitudinal sections. The paraffin-embedded sections were stained with hematoxylin and eosin (H&E) according to the manufacturer’s instructions (Beyotime, Shanghai, China). All images were acquired using a light microscope (IX71; Olympus, Tokyo, Japan).

### Immunohistochemistry

Immunohistochemical staining procedures were conducted as described [42]. The antibodies used were listed as follows: anti-EGFR (Cat. #4267, Cell Signaling Technology; 1:50), anti-phosphorylated EGFR (Cat. #3777, Cell Signaling Technology;1:200), anti-NEDD4L (Cat. #ab46521, Abcam; 1:200), anti-FOXM1 (Cat. #ab207298, Abcam; 1:200), and anti-Ki67 (Cat. #ab16667, Abcam; 1:200). Images were captured and analyzed using a light microscope (IX71; Olympus, Tokyo, Japan). The immunostaining staining score (H-score) was carried out according to a previous study [43]. Quantification of Ki67 staining were assessed by percentage of positive staining cells as previously described [44].

### Cell viability assay

Cell viability was measured by CCK8 kit (Dojindo Laboratories, Kumamoto, Japan). Briefly, cells were seeded at a density of 3 × 10^3^ cells per well in 96-well plates and incubated overnight. Cells were treated with indicated doses of inhibitors and then cultured for designated Durations. 10 μL of CCK-8 solution (Dojindo Laboratories, Kumamoto, Japan) was added to each well and incubated continuously for an additional 2 h. Finally, the absorbance value (at OD = 450 nm) was detected.

### 5-Ethynyl-2’-deoxyuridine (EdU) assay

EdU assay was undertaken with BeyoClick™ EdU Cell Proliferation Kit with Alexa Fluor 594 (Beyotime, Shanghai, China). Briefly, cells were plated in confocal dishes (NEST Biotechnology) and incubated overnight. Then cells were treated with indicated doses of inhibitors and cultured for designated durations. According to the manufacturer’s instructions, after washing in PBS, cells were incubated with EdU solution for 2 h. Cell nuclei were subsequently labeled by using Hoechst 33,342 (Beyotime, Shanghai, China) for 5 min. Staining images were captured under a confocal fluorescence microscope (ZEISS,Jena,Germa).

### Colony formation assay

Cells were seeded at a density of 500–1000 per well in 12-well plates and incubated overnight. Cells were treated with indicated doses of inhibitors and then cultured for 7–10 days to allow visible clones appeared. Cell clones were fixed in 4% paraformaldehyde and stained with 1% crystal violet. Finally, the cells were photographed and clones containing more than 50 cells were counted.

### Western blot and CO-immunoprecipitation (CO-IP) assay

The cells or tumor tissues were harvested and then lysed in cold radioimmunoprecipitation assay (RIPA) buffer (Beyotime, Shanghai, China) containing protease/phosphatase inhibitor cocktail for 30 min. The clarified supernatants were collected after centrifugation (12,000 × g, 20 min) and quantified using a BCA protein assay (Beyotime, Shanghai, China). Subsequently, equal amounts of protein lysates in each group were loaded and separated by SDS-PAGE, transferred onto PVDF membranes, blocked with 5% non-fat milk, probed with corresponding primary antibodies and HRP-conjugated anti-rabbit or mouse IgG secondary antibody, and finally visualized using an enhanced chemiluminescence system (BioRad; Hercules, CA, USA).

For Co-IP, equal amounts of total protein lysates were first incubated with anti-Flag-magnetic Beads (Beyotime, Shanghai, China), anti-HA-magnetic Beads (Beyotime, Shanghai, China), or anti-His-magnetic Beads (Beyotime, Shanghai, China) for 4 h at 4 °C. Beads were applied to a magnet and washed six times using with cold RIPA buffer (Beyotime, Shanghai, China). The proteins associated with beads were eluted and subjected to western blotting.

### Animal study

The experimental procedures involving mice were conducted in accordance with the guidelines of the Institutional Animal Care and Use Committee (IACUC) of the Sun Yat-sen University Cancer Center. The study adhered to all relevant guidelines and regulations outlined in the approved protocol.

For cell line-derived xenograft (CDX) model, female BALB/c nude mice (4- or 5-week-old, Weighing 14-16g) were procured from Animal Research Center of Sun Yat-sen University Cancer Center and maintained under specific pathogen-free condition. Indicated cells (5 × 10^6^/200 μl) were subcutaneously injected into the right flank of each mouse (five mice per group). For drugs treatment assays, when the tumor grows approximately 100 mm^3^, the mice were received indicated inhibitors alone or combination, including verteporfin (30 mg/kg [45] in PBS containing 10% DMSO and 45% PEG 300 and 5% Tween-80, i.p. once daily), osimertinib (5 mg/kg in PBS containing 10% DMSO, 40% PEG 300, and 5% Tween-80 by gastric gavage once daily). During the experiment, the body weight and tumor size of each mouse were measured every three days. The tumor volume (V) was estimated using the formula: V = L(length) × W (width)^2^/2. The mice were euthanized at the end of the study, and their tumors were excised, photographed, weighed and harvested.

For genetically engineered mouse model, a doxycycline-inducible EGFR^19del/T790M^ transgenic model was kindly provided as a gift from Professor Liang Chen (College of Life Science and Technology, Jinan University, Guangzhou, China). Specifically, the clara cell 10-kD protein (CC10) promoter and the reverse tetracycline transactivator (rtTA) were used to create a lung-specific, externally regulatable, overexpression transgenic system. Age-matched and sex-matched transgenic mice on FVB background were used in the experiments. Transgene expression was activated at 4–5 weeks of age for the induction of tumors. Mice with double positive EGFR^19del/T790M^ & CC10rtTA were administered doxycycline in feedstuff throughout the study. The animal model was established using a slightly modified version of a previously described method [42]. For drugs treatment assays, when transgene expression was activated for six weeks, the mice (*n* = 3 per group) were received indicated inhibitors alone or combination, including verteporfin (20 mg/kg in PBS containing 10% DMSO and 45% PEG 300 and 5% Tween-80, i.p. once daily), osimertinib (2 mg/kg in PBS containing 10% DMSO, 40% PEG 300, and 5% Tween-80 by gastric gavage once daily), FDI-6 (50 mg/kg [46] in PBS containing 10% DMSO and 45% PEG 300 and 5% Tween-80, i.p. once daily). The mice were euthanized at the end of the study, and their lungs were excised, photographed, weighed and harvested.

### Statistical analysis

Statistical analysis was carried out by using Graph Pad Prism 8 (San Diego, CA, USA) and SPSS 20.0 software (SPSS Inc., Chicago, IL, USA). All numerical data were expressed as mean ± standard deviation (SD). Experiments were carried out with three or more replicates. Two or more groups were assessed by using Student’s t test or ANOVA individually as well as Kruskal Wallis Test, Mann–Whitney Test. Correlation analysis was analyzed using Pearson correlation. Survival was analyzed employing the Kaplan–Meier method with the log-rank test. *P* value < 0.05 was considered as statistically significance.

## Results

### Patients harboring EGFR amplification derive less clinical benefit from third-generation EGFR-TKIs as first-line treatment

**Fig. 1:**
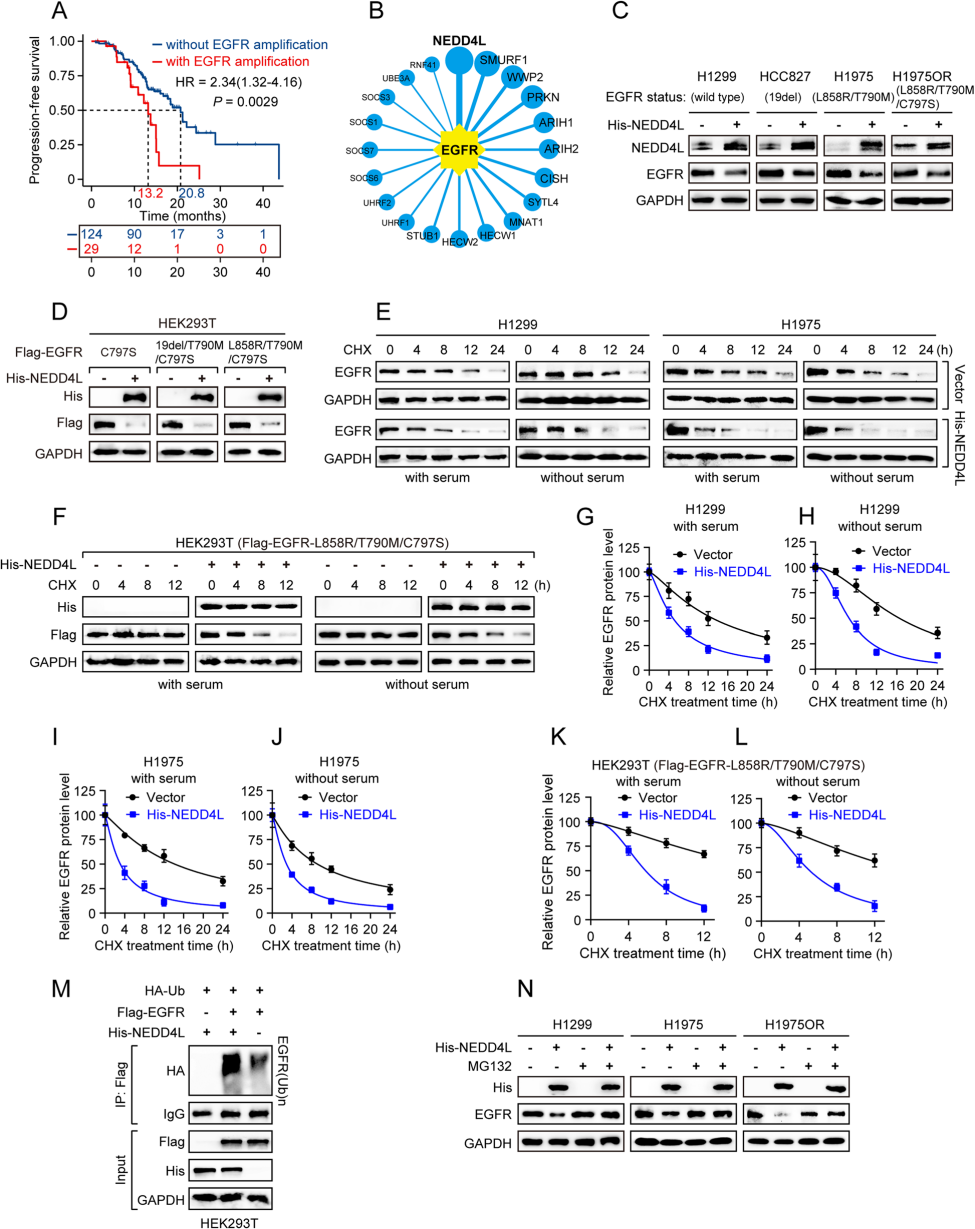
NEDD4L promotes EGFR protein ubiquitination and proteasome-mediated degradation. **A** A total of 153 EGFR-mutant LUAD patients treated with the third-generation EGFR-TKIs as the first-line treatment. The Progress-free survival curves of these patients in with and without EGFR amplification group. **B** Top 20 E3 ubiquitin ligases of EGFR predicted by UbiBrowser database. **C** Western blot to detect the protein level of EGFR in LUAD cells (H1299, HCC827, H1975, H1975OR) stably expressing NEDD4L. **D** HEK293T cells were co-transfected with Flag-EGFR (bearing C797S only, or 19del/T790M/C797S, or L858R/T790M/C797S mutations) and His-NEDD4L expressing plasmids for 36 h, followed by western blot analyses. **E** H1299 or H1975 cells stably expressing His-NEDD4L were incubated in the absence or presence of serum (10%) for 12 h, and then treated with cyclohexamide (CHX) (50 µg/mL) for indicated time periods, followed by western blot analyses. **F** HEK293T cells were co-transfected with Flag-EGFR (bearing L858R/T790M/C797S mutations) and His-NEDD4L expressing plasmids for 36 h, and then treated with cyclohexamide (CHX) (50 µg/mL) for indicated time periods, followed by western blot analyses. **G**-**L** Quantitative analysis of (**E**, **F**) results. **M** HEK293T cells were co-transfected with HA-ubiquitin, Flag-EGFR, and either His-NEDD4L expressing plasmids for 36 h, and then treated with MG132 (20 μM) for 4 h, followed by IP-Western analyses. **N** H1299, H1975 or H1975OR cells stably expressing His-NEDD4L were treated with MG132 (20 μM) for 6 h, followed by western blot analyses.

A total of 153 advanced LUAD patients, harboring EGFR active mutations (L858R and 19del), were enrolled in this analysis (Supplementary Table S1). All patients received the third-generation EGFR-TKIs as the first-line treatment (including osimertinib, aumolertinib and furmonertinib). Based on the copy number of EGFR, the patients were categorized into two groups: EGFR-amplification group (19%) and EGFR-non-amplification group (81%). Notably, patients in EGFR-amplification group exhibited significantly shorter duration of PFS compared to those in non-amplification group (13.2m *vs.* 20.8m; log-rank *P* = 0.0029) (Fig. 1A), indicating EGFR amplification undermines the efficacy of third-generation EGFR-TKIs in EGFR-mutated LUAD. Notably, increasing number of studies have consistently highlighted that EGFR amplification is one of the target-dependent mechanisms contributing to EGFR-TKIs resistance [47–49]. These findings including ours suggest that combination therapy incorporating EGFR expression blockade may be a promising therapeutic strategy against EGFR-mutated LUAD, both in post-TKI resistance and upfront treatment settings, worthy of further study.

### NEDD4L promotes EGFR protein ubiquitination and proteasome-mediated degradation

In addition to EGFR amplification, dysregulation of EGFR degradation is also a crucial determinant in accelerating lung cancer initiation and progression, even in mutant EGFR-driven LUAD [13, 42, 50], emphasizing that facilitating EGFR protein degradation is a promising replacement or combination strategy for the treatment of EGFR-driven LUAD. Thus, to identify the pivotal factors regulating EGFR degradation, we first employed the UbiBrowser 2.0 online database to predict the potential E3 ubiquitin Ligases that target EGFR for degradation. The top 20 predicted E3 ubiquitin ligases were listed in Fig. 1B, of which we chose the top eight predicted E3 ubiquitin ligases for further experimental validation. Notably, among these, only NEDD4L knockdown significantly upregulated EGFR protein expression in multiple LUAD cell lines, including H1299 cells (harboring wild-type EGFR), H1975 cells (harboring EGFR^L858R/T790M^) and H1975OR cells (harboring EGFR^L858R/T790M/C797S^) (Supplementary Fig. S1A-C).

To further validate the regulatory role of NEDD4L on EGFR, we overexpressed NEDD4L in several LUAD cell lines, including H1299 cells, HCC827 cells (harboring EGFR^19del^), H1975 cells and H1975OR cells. Our results showed that NEDD4L overexpression can not only downregulate the protein expression of wild-type EGFR, but also its mutants, including EGFR^L858R/T790M/C797S^, which confers osimertinib resistance (Fig. 1C), without any alteration in mRNA level (Supplementary Fig. S1D). Additionally, ectopic expression of NEDD4L also led to the downregulation of EGFR^C797S^, EGFR^19del/T790M/C797S^ or EGFR^L858R/T790M/C797S^ mutant protein in HEK293T cells (Fig. 1D), all of which are known mediators of osimertinib resistance, suggesting a potential role for NEDD4L in regulating osimertinib resistance. Furthermore, we found that NEDD4L^WT^ overexpression could decrease EGFR protein level, whereas NEDD4L^Mut^ (C942A, E3 ubiquitin ligase active site) failed (Supplementary Fig. S1E), indicating that the inhibitory effect of NEDD4L on EGFR protein expression is dependent on its E3 ubiquitin ligase activity. Despite previous studies have reported that EGFR undergoes rapid destabilization upon EGF stimulation [14, 15], our results showed that NEDD4L effectively destabilized EGFR protein, regardless of the presence or absence of EGF (Supplementary Fig. S1F), indicating that NEDD4L-mediated EGFR protein inhibition is independent of EGF stimulation.

We next examined whether NEDD4L acts as a reliable E3 ubiquitin ligase for EGFR. As seen in Fig. 1E-L, NEDD4L overexpression significantly shortened the half-life of wild-type EGFR, EGFR^L858R/T790M^ and EGFR^L858R/T790M/C797S^ proteins, irrespective of the presence or absence of serum. Besides, overexpression of NEDD4L promoted ubiquitination of EGFR protein (Fig. 1M). Notably, the reduction of EGFR protein levels mediated by NEDD4L was effectively reversed by proteasome inhibitor MG132 in H1299, H1975 and H1975OR cells (Fig. 1N), but not by lysosome inhibitor chloroquine (CLQ) (Supplementary Fig. S1G). Taken together, these results suggest that NEDD4L destabilizes EGFR (including wild-type EGFR, osimertinib-sensitive/resistant EGFR) protein through ubiquitination and proteasome-mediated degradation.

### NEDD4L interacts with EGFR

**Fig. 2:**
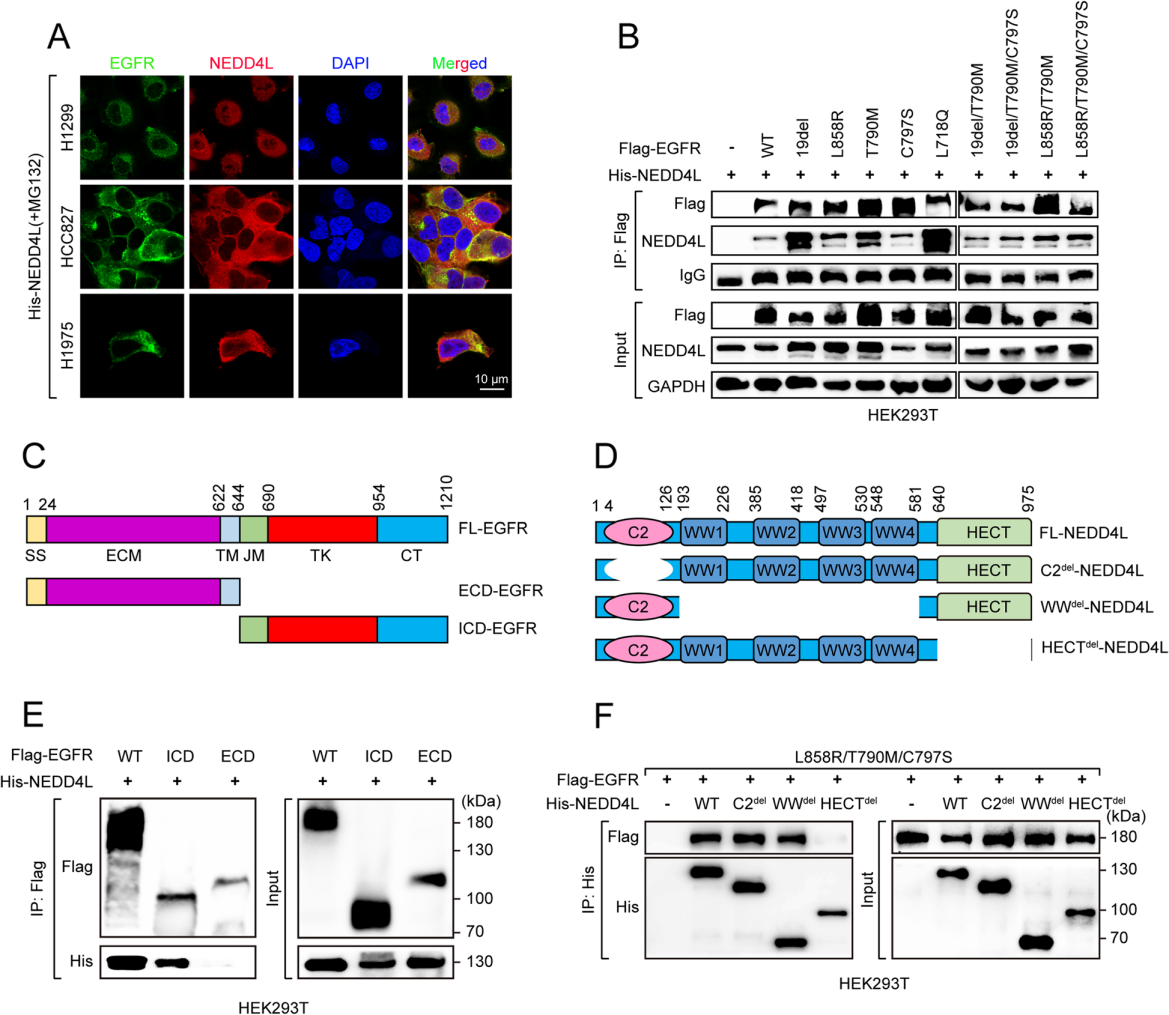
NEDD4L interacts with EGFR. **A** H1299, HCC827 or H1975 cells were transfected with His-NEDD4L expressing plasmids for 36 h, and then treated with MG132 (20 μM) for 6 h, followed by Immunofluorescence assays of NEDD4L (red) and EGFR (green) and DAPI (blue). Scale bar = 10 µm. **B** HEK293T cells were co-transfected with His-NEDD4L and indicated Flag-EGFR-mutant expressing plasmids for 36 h, and then treated with MG132 (20 μM) for 6 h, followed by IP-Western analyses. **C** Schematic representation of EGFR truncated mutants. FL, full length; ICD, intracellular domain; ECD, extracellular domain. **D** Schematic representation of NEDD4L truncated mutants. Del, deletion. **E** HEK293T cells were co-transfected with His-NEDD4L and indicated truncated Flag-EGFR-mutant expressing plasmids for 36 h, and then treated with MG132 (20 μM) for 6 h, followed by IP-Western analyses. **F** HEK293T cells were co-transfected with Flag-EGFR and indicated truncated His-NEDD4L-mutant expressing plasmids for 36 h, and then treated with MG132 (20 μM) for 6 h, followed by IP-Western analyses.

Next, to address the mechanism of NEDD4L in destabilizing EGFR protein, we did immunofluorescence assay and found colocalization of NEDD4L with wild-type EGFR, EGFR^19del^ or EGFR^L858R/T790M^ in H1299, HCC827 and H1975 cells, respectively (Fig. 2A). Moreover, CO-immunoprecipitation (CO-IP) analysis showed that NEDD4L binds to not only wild-type EGFR protein, but also multiple osimertinib-sensitive/resistant EGFR mutant proteins, including EGFR^19del^, EGFR^L858R^, EGFR^T790M^, EGFR^C797S^, EGFR^L718Q^, EGFR^19del/T790M^, EGFR^19del/T790M/C797S^, EGFR^L858R/T790M^ or EGFR^L858R/T790M/C797S^ (Fig. 2B). To dissect the potential binding domains of EGFR and NEDD4L, we employed ZDOCK 3.0.2, a widely utilized tool for predicting protein–protein interaction. Our results indicated that NEDD4L and EGFR may interact through eight potential amino acid residues (Ser991, Asp807, Tyr801, Met793, His988, Leu989, Lys716, Lys713) located on the interface of the EGFR-intracellular domain (EGFR-ICD) and nine (Tyr853, Arg918, Trp910, Arg925, Asn900, Asn851, Gly852, Gln764, Gln760) on the interface of the NEDD4L-HECT domain (Supplementary Fig. S2A, B). Further CO-IP assay confirmed that both the EGFR-ICD domain and NEDD4L-HECT domain were essential for NEDD4L-EGFR interaction (Fig. 2C-F). These findings suggest that NEDD4L could interact with EGFR, either wild-type EGFR or its actionable and resistant mutants, highlighting the potential significance of this interaction in destabilizing EGFR protein.

### NEDD4L inhibits LUAD growth through decreasing EGFR protein expression

**Fig. 3:**
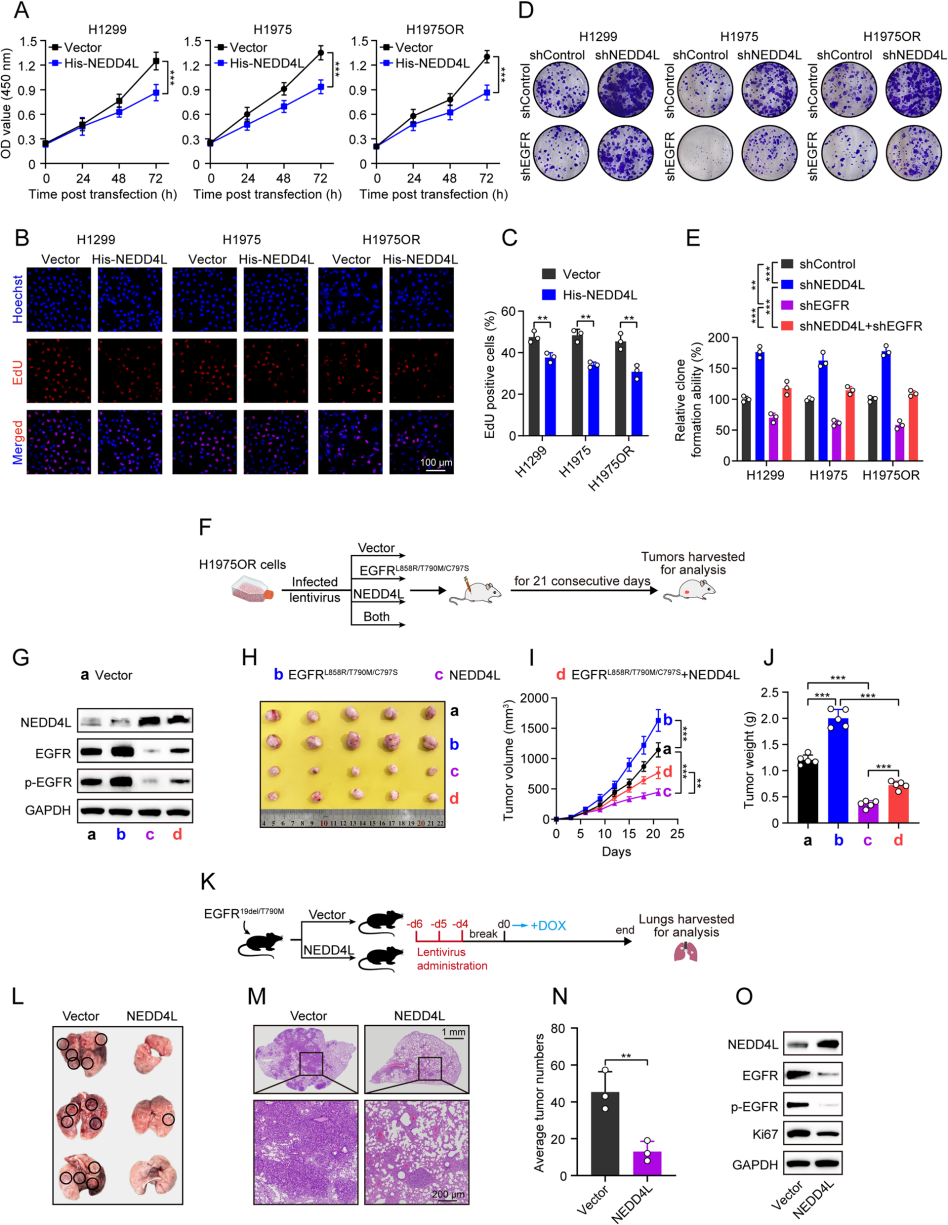
NEDD4L inhibits LUAD cell proliferation through decreasing EGFR protein expression. **A**-**B** H1299, H1975 or H1975OR cells stably expressing either vector or His-NEDD4L were incubated for indicated time periods, followed by (**A**) CCK8 assays, or (**B**) EdU assays. Scale bar = 100 µm. **C** Quantitative analysis of (**B**) results. **D** H1299, H1975 or H1975OR cells stably expressing shNEDD4L, or shEGFR, or both were incubated for 10 days, followed by colony formation assays. **E** Quantitative analysis of (**D**) results. **F** Experimental design of CDX model. H1975OR cells (bearing EGFR^L858R/T790M/C797S^ mutations) stably expressing either Flag-EGFR^L858R/T790M/C797S^, or His-NEDD4L, or both were injected subcutaneously in female BALB/c nude mice (*n* = 5/group) for inoculation of 21 days, then tumors were harvested for analysis. **G** Western blot analyses of NEDD4L, EGFR and phosphorylated EGFR protein expression in final shaped tumors. **H** Photos of final shaped tumors. **I** Tumor growth curve. **J** Final tumor weight. **K** Experimental design of transgenic mice model. Female EGFR^19del/T790M^ transgenic mice (*n* = 3/group) were intranasally administrated with vector or NEDD4L lentivirus once Daily for 3 consecutive days. One week after the first lentiviral administration, DOX was added to feed the mice for 6 weeks, then the lung samples were harvested for analysis. **L** Photos of final lung samples. **M** H&E staining of final lung samples. Scale bar = 1mm (up) or 200 µm (down). **N** Numbers of observable nodules in the final lung surface. **O** Western blot analyses of NEDD4L, EGFR, phosphorylated EGFR and Ki67 protein expression in final lung nodule tissues. ^**^P < 0.01, ^***^P < 0.001.

The above results prompted us to investigate the functional role of the NEDD4L/EGFR axis in LUAD. As seen in Fig. 3A-C, NEDD4L overexpression significantly inhibited cell proliferation of H1299, H1975 and H1975OR cells. Conversely, NEDD4L knockdown promoted cell proliferation (Supplementary Fig. S3A-F). EGFR knockdown reversed the pro-proliferation effect induced by NEDD4L knockdown in H1299, H1975 and H1975OR cells (Fig. 3D, E). Consistently, in vivo growth assay of H1975OR cell-derived xenografts showed that NEDD4L overexpression exhibited significantly decreased tumor growth rates compared with the vector group (Fig. 3F-J). Moreover, NEDD4L overexpression also reversed the tumor growth enhancement caused by EGFR^L858R/T790M/C797S^ overexpression in H1975OR cells (Fig. 3F-J), which also represented EGFR amplification states, indicating that NEDD4L can inhibit proliferation of LUAD cells driven not only by EGFR mutants but also EGFR amplification. To further validate our findings, we established a doxycycline (DOX)-inducible EGFR^19del/T790M^ transgenic mice model. Mice were treated with NEDD4L-overexpressed lentivirus for three consecutive days, starting six days prior to DOX administration to activate the mutant EGFR transgene (Fig. 3K). The results showed that DOX-induced EGFR^19del/T790M^ expression effectively drove lung tumor formation, which was significantly suppressed by lentiviral expression of NEDD4L as evidenced by tumor indexes and H&E staining of tumor sections (Fig. 3L-N), as well as proliferation marker of Ki67 expression (Fig. 3O; Supplementary Fig. S3G, H). Furthermore, lung lesions derived from mice of NEDD4L-overexpressed group exhibited a significant reduction in EGFR and p-EGFR protein expression (Fig. 3O; Supplementary Fig. S3I).

Collectively, these results suggest that NEDD4L inhibits LUAD growth through decreasing EGFR protein expression.

### FOXM1 transcriptionally inhibits NEDD4L to promote tumor growth through increasing EGFR protein expression in EGFR-driven LUAD

**Fig. 4:**
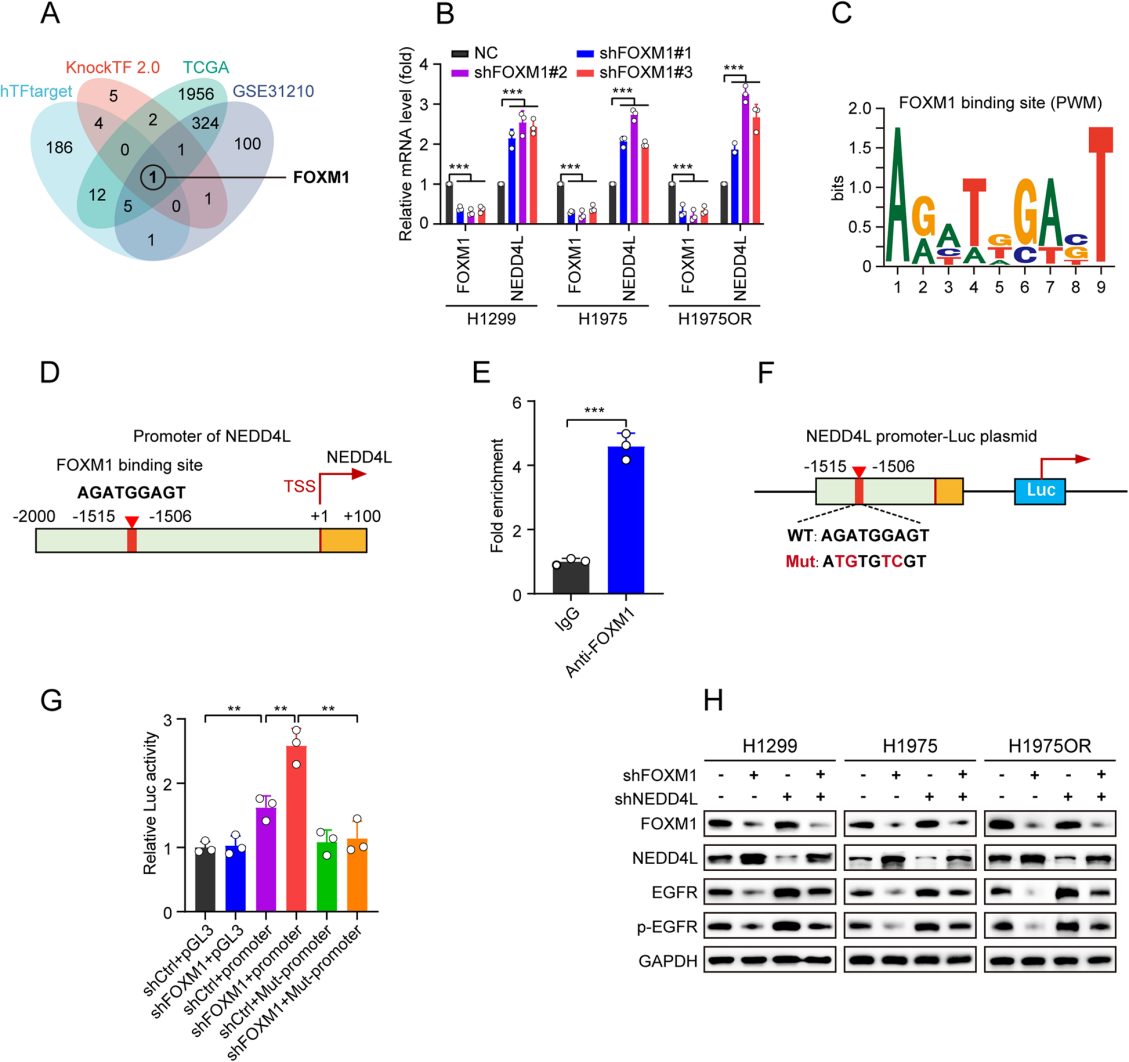
FOXM1 directly binds to NEDD4L promoter and represses its expression to increase EGFR protein level. **A** Prediction of potential upstream transcription factors of NEDD4L in LUAD. Four approaches including hTFtarget and KnockTF 2.0 database to identify potential upstream transcription factors of NEDD4L and TCGA-LUAD cohort and GEO dataset (GSE31210) to search for NEDD4L-related DEGs. **B** H1299, H1975, or H1975OR cells stably expressing either shControl or shFOXM1 were incubated for 24 h, followed by qPCR analyses. **C** Map of the position weight matrix (PWM) for FOXM1 binding motif sequence from MotifMap database. **D** Schematic illustration of the predicted FOXM1 binding site in the NEDD4L promoter using hTFtarget. TSS,transcriptional start site. **E** ChIP analysis of FOXM1 binding to the NEDD4L promoter in H1299 cells. **F** Schema of the putative FOXM1 binding site in the promoter of NEDD4L, the consensus and mutant sequences for FOXM1 binding were boxed. **G** H1299 cells stably expressing either shControl or shFOXM1 were co-transfected with NEDD4L promoter-wt or -mutant plasmids for 36 h, followed by dual luciferase reporter assay. **H** H1299, H1975 or H1975OR cells stably expressing shFOXM1, shNEDD4L, or both were incubated for 36 h, followed by western blot analyses. ^**^P < 0.01, ^***^P < 0.001.

Given the pivotal role of NEDD4L in EGFR-driven LUAD growth, we sought to investigate how NEDD4L is downregulated. We performed in silico analyses using the hTFtarget and KnockTF 2.0 databases to identify transcription factors that potentially regulating NEDD4L and TCGA-LUAD and GEO dataset (GSE31210) to search for NEDD4L-related differentially expressed genes (DEGs). By intersecting the results from these approaches, we discovered that FOXM1 was a potential transcription factor regulating NEDD4L (Fig. 4A). Subsequent experiments in H1299, H1975 and H1975OR cells showed that FOXM1 knockdown significantly upregulated the mRNA expression of NEDD4L (Fig. 4B). To further explore whether FOXM1 directly regulates NEDD4L, we first identified a region for FOXM1 located within − 1515 to − 1506 base pairs of the NEDD4L transcriptional start site by bioinformatics approach (Fig. 4C, D) and then validated the specific binding of FOXM1 to the NEDD4L promoter by chromatin immunoprecipitation (ChIP) assay (Fig. 4E). Dual luciferase assay further showed that mutations in this region substantially abolished the luciferase activity (Fig. 4F, G). These results indicated that FOXM1 directly binds to the promoter of NEDD4L and represses its expression. Moreover, both FOXM1 knockdown and treatment with FDI-6, a known FOXM1 inhibitor used in scientific research, significantly increased NEDD4L protein expression and decreased the expression of wild-type EGFR, EGFR^L858R/T790M^, and EGFR^L858R/T790M/C797S^ protein in H1299, H1975 and H1975OR cells, respectively (Supplementary Fig. S3J, K). Notably, NEDD4L knockdown reversed the decrease in EGFR and p-EGFR protein level caused by FOXM1 knockdown (Fig. 4H). These results indicated that FOXM1 is a critical upstream transcription factor that binds to the promoter of NEDD4L and represses its expression, thus increasing EGFR protein expression.

**Fig. 5:**
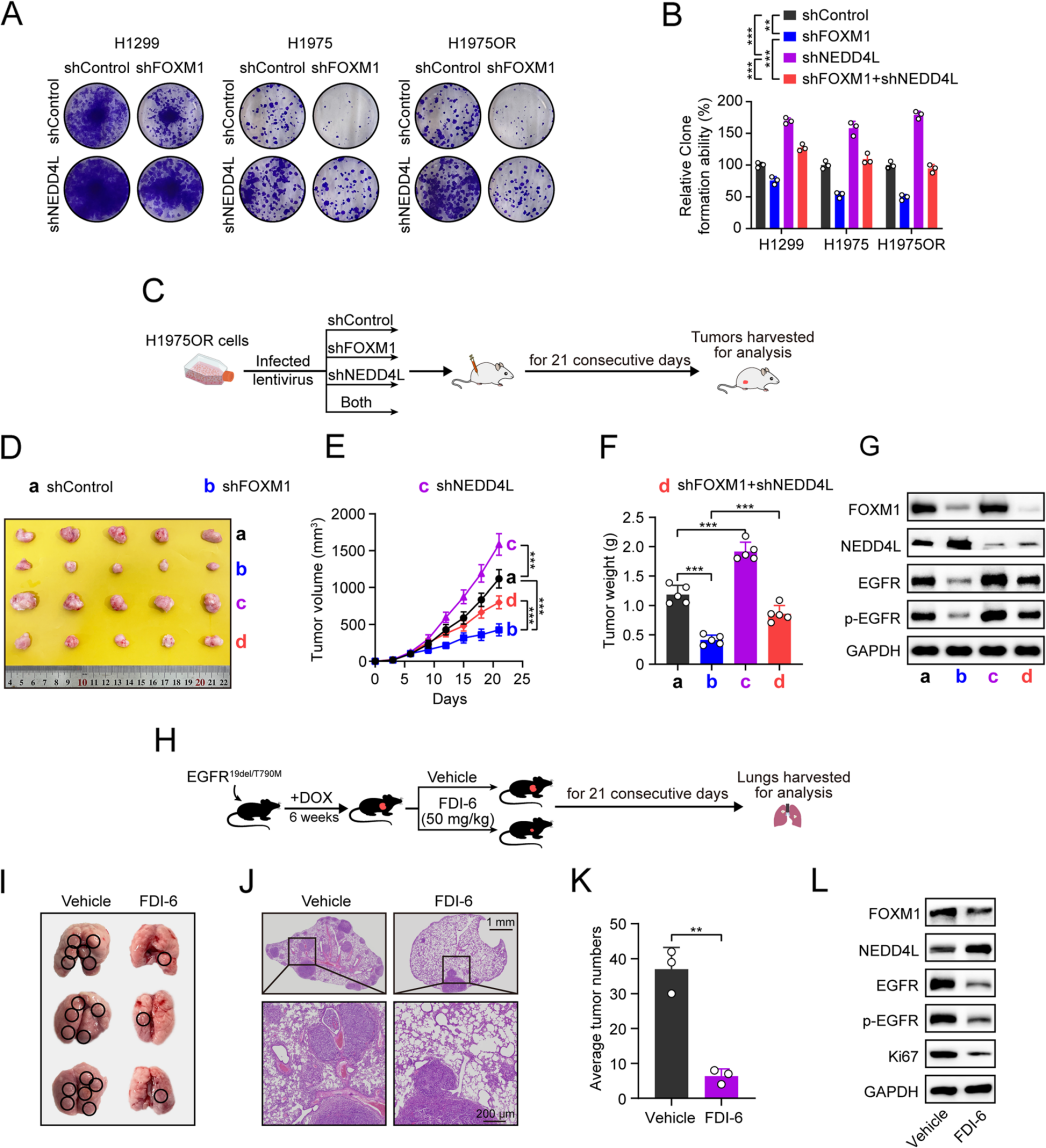
FOXM1 promotes EGFR-driven LUAD growth through inhibiting NEDD4L expression. **A** H1299, H1975 or H1975OR cells stably expressing shFOXM1, shNEDD4L, or both were incubated for 10 days, followed by colony formation assays. **B** Quantitative analysis of (**A**) results. **C** Experimental design of CDX model. H1975OR cells (bearing EGFR^L858R/T790M/C797S^ mutations) stably expressing either shFOXM1, or shNEDD4L, or both were injected subcutaneously in female BALB/c nude mice (*n* = 5/group) for inoculation of 21 days, then tumors were harvested for analysis. **D** Photos of final shaped tumors. **E** Tumor growth curve. **F** Final tumor weight. **G** Western blot analyses of FOXM1, NEDD4L, EGFR, phosphorylated EGFR and Ki67 protein expression in final shaped tumors. **H** Experimental design of transgenic mice model. DOX-induced female EGFR^19del/T790M^ transgenic mice (*n* = 3/group) were intraperitoneally treated with FOXM1 inhibitor FDI-6 (50 mg/kg) Daily for a period of 21 days, then the lung samples were harvested for analysis. **I** Photos of final lung samples. **J** H&E staining of final lung samples. Scale bar = 1mm (up) or 200 µm (down). **K** Numbers of observable nodules in the final lung surface. **L** Western blot analyses of FOXM1, NEDD4L, EGFR, phosphorylated EGFR and Ki67 protein expression in final lung nodule tissues. ^**^P < 0.01, ^***^P < 0.001.

We subsequently investigated whether FOXM1 regulates LUAD growth through inhibition of NEDD4L. As shown in Fig. 5A and B, FOXM1 knockdown significantly inhibited LUAD cell proliferation, and such decrease was reversed by knockdown of NEDD4L in H1299, H1975 and H1975OR cells. Moreover, the in vivo growth assays of H1975OR cell-derived xenografts showed that FOXM1 knockdown significantly suppressed tumor growth, whereas NEDD4L knockdown promoted tumor growth compared with the control group. Moreover, NEDD4L knockdown restored the tumor growth suppression induced by FOXM1 knockdown (Fig. 5C-F). Further western blot analysis showed that NEDD4L knockdown reversed the EGFR inactivation induced by FOXM1 knockdown (Fig. 5G). Similarly, in a DOX-inducible EGFR^19del/T790M^ transgenic mice model (Fig. 5H), the in vivo growth assays showed that FOXM1 inhibitor FDI-6 significantly inhibited tumor growth compared to the control group (Fig. 5I-K). Western blot and IHC analyses showed that FDI-6 exhibited a significant reduction in FOXM1, EGFR and p-EGFR expression, along with increased NEDD4L expression (Fig. 5L; Supplementary Fig. S3L).

Taken together, these findings suggest that FOXM1 transcriptionally represses NEDD4L to increase EGFR protein expression, thus promoting tumor growth in EGFR-driven LUAD.

### High FOXM1 expression correlates with low NEDD4L expression in LUAD patients, which is associated with poor clinical outcomes

**Fig. 6:**
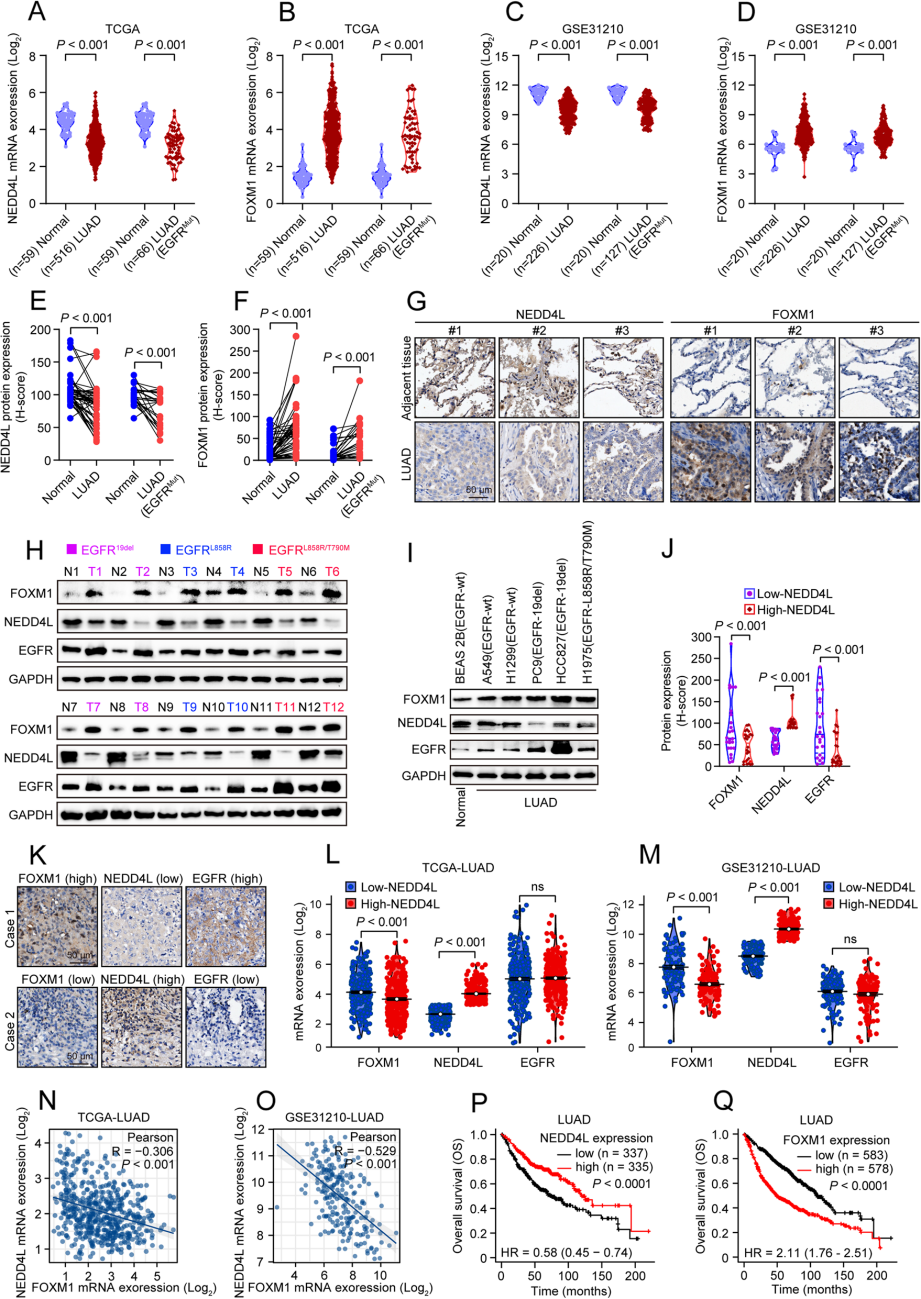
Both high-FOXM1 and low-NEDD4L expression predict poor clinical outcomes of LUAD patients. **A**-**B** The comparative expression of NEDD4L(**A**) and FOXM1(**B**) mRNA between LUAD and normal tissues in TCGA database. **C**-**D** The comparative expression of NEDD4L(**C**) and FOXM1(**D**) mRNA between LUAD and normal tissues in GSE31210 dataset. **E**–**F** The comparison expression of NEDD4L (**E**) and FOXM1 (**F**) protein between LUAD and paired normal tissues from tissue microarray slides for IHC analyses. Staining was quantified by H-score. **G** Representative images of IHC staining. Scale bar = 50 µm. **H** Western blot analyses of FOXM1, NEDD4L and EGFR protein in twelve pairs of LUAD and adjacent samples collected from our cancer center. **I** Western blot analyses of FOXM1, NEDD4L and EGFR protein expression level in LUAD cell lines (A549, H1299, PC9, HCC827, H1975) and the normal lung epithelial cell line (BEAS-2B). **J** The comparison of FOXM1, NEDD4L and EGFR protein level in low- and high-NEDD4L expression groups from LUAD tissue microarray slides for IHC analyses. **K** Representative images of IHC staining. Scale bar = 50 µm. **L**-**M** The comparative expression of FOXM1, NEDD4L and EGFR mRNA level in low- and high-NEDD4L expression groups in TCGA database (**L**) and GSE31210 dataset (**M**). ns, not significant. **N**–**O** The expression correlation of FOXM1 and NEDD4L in LUAD from TCGA (**N**) database and GSE31210 (**O**) dataset. **P** Kaplan–Meier plots of overall survival (OS) of LUAD patients with high or low expression of NEDD4L. **Q** Kaplan–Meier plots of overall survival (OS) of LUAD patients with high or low expression of FOXM1.

The above results prompted us to further investigate the clinical relevance of FOXM1, NEDD4L and EGFR in LUAD. Analysis of TCGA-LUAD cohort at the mRNA level revealed abnormally low expression of NEDD4L and high expression of FOXM1 in LUAD and even EGFR-mutated LUAD (Fig. 6A-B), which was further verified by analysis of the GSE31210 dataset (Fig. 6C-D). In line with the above results, IHC analysis of tissue microarrays (TMA) showed the same expression pattern of FOXM1 and NEDD4L at protein level in LUAD and even EGFR-mutated LUAD (Fig. 6E-G). Notably, when comparing twelve clinical LUAD tissues harboring EGFR mutants (19del, L858R, L858R/T790M) to their matched adjacent tissues, we observed reduced NEDD4L expression and increased FOXM1 and EGFR expression (Fig. 6H). Comparable results were also observed in LUAD cell lines carried either wild-type EGFR or EGFR mutants in comparison to the normal pulmonary epithelial cell line (Fig. 6I). Additional analysis of TMA further showed that LUAD patients with higher protein expression level of NEDD4L tended to have lower FOXM1 and EGFR protein expression levels, and vice versa (Fig. 6J, K). While at mRNA level, it was found FOXM1, not EGFR, that negatively correlated with NEDD4L expression in LUAD (Fig. 6L, M). Further correlation analysis of TCGA and GSE31210 datasets confirmed that FOXM1 was negatively correlated with NEDD4L expression in LUAD (Fig. 6N, O). Furthermore, Kaplan–Meier survival analysis showed that high-FOXM1 and low-NEDD4L expression were significantly associated with poor overall survival (OS) in LUAD (Fig. 6P, Q).

Collectively, these results suggest the close correlation among FOXM1, NEDD4L and EGFR expression in LUAD, and both high FOXM1 expression and low NEDD4L are associated with poor clinical outcomes.

### Verteporfin is a novel inhibitor of FOXM1 in facilitating NEDD4L-mediated EGFR proteasomal degradation and inhibiting LUAD growth

**Fig. 7:**
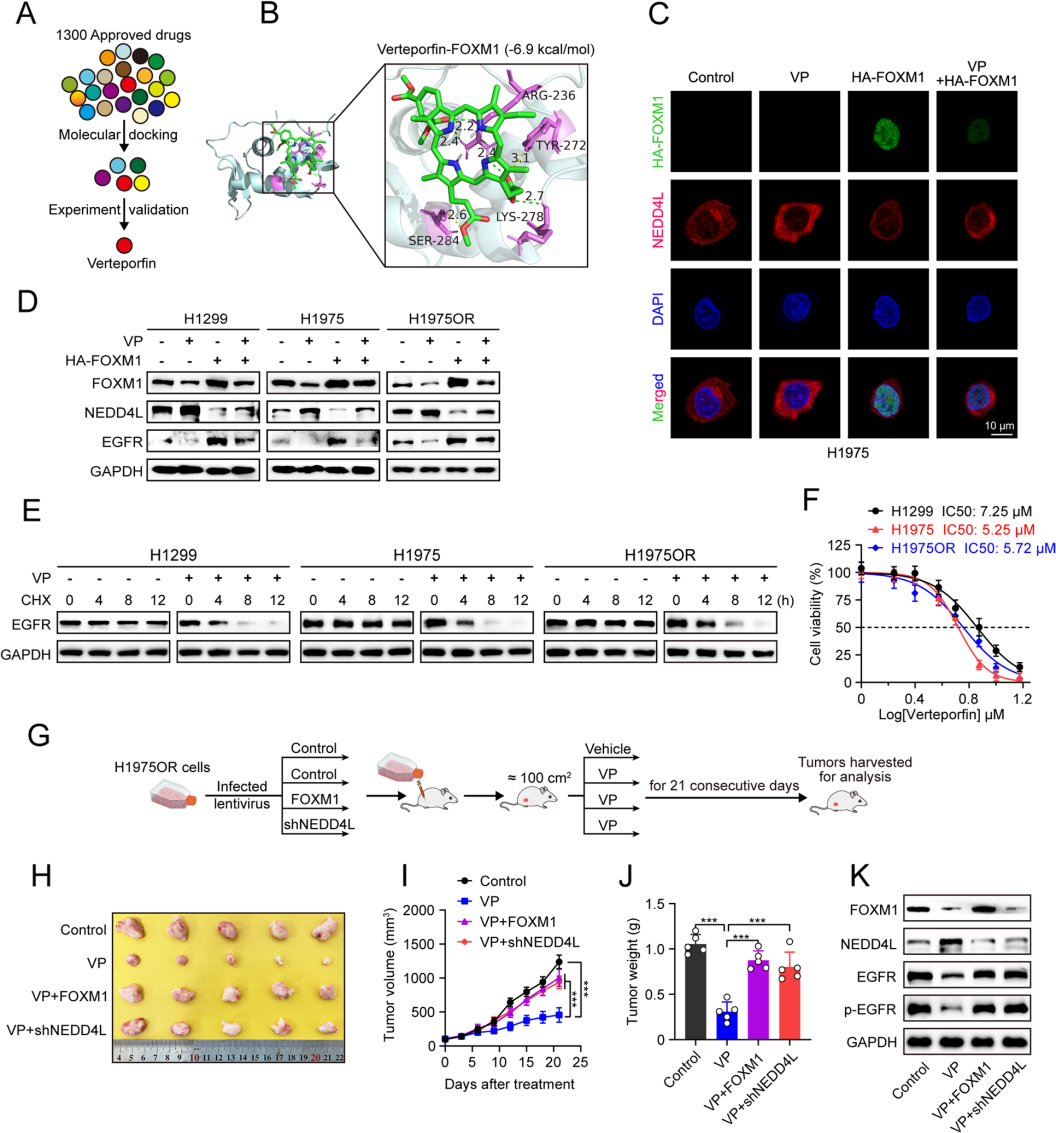
Verteporfin is a novel inhibitor of FOXM1 in facilitating NEDD4L-mediated EGFR proteasomal degradation and inhibiting LUAD growth. **A** Flow chart showing the strategy used to screen FOXM1 inhibitors. **B** Molecular docking diagram of the interaction between FOXM1 protein and verteporfin. Purple represents FOXM1, and green represents verteporfin. **C** H1975 cells stably expressing empty vector or HA-FOXM1 were treated with verteporfin for 36 h, followed by Immunofluorescence staining. Scale bar = 10 µm. **D** H1299, H1975 or H1975OR cells stably expressing empty vector or HA-FOXM1 were treated with verteporfin for 36 h, followed by western blot analyses. **E** H1299, H1975 or H1975OR cells were firstly treated with or without verteporfin for 24 h, then added cyclohexamide (CHX) (50 µg/mL) to co-incubate for indicated time periods, followed by western blot analyses. **F** H1299, H1975 or H1975OR cells were treated with indicated doses of verteporfin for 48 h, followed by CCK8 assays. **G** Experimental design of CDX model. H1975OR cells (bearing EGFR^L858R/T790M/C797S^ mutations) stably expressing either FOXM1 or shNEDD4L were injected subcutaneously in female BALB/c nude mice (*n* = 5/group) for inoculation. When the tumor size reach about 100 mm^3^, mice were intraperitoneally treated with verteporfin (30 mg/kg) Daily for a period of 21 days, then the tumors were harvested for analysis. **H** Photos of final shaped tumors. **I** Tumor growth curve. **J** Final tumor weight. **K** Western blot analyses of FOXM1, NEDD4L, EGFR and phosphorylated EGFR protein expression in final shaped tumors. VP, verteporfin. ^***^P < 0.001.

Given the pivotal role of the FOXM1/NEDD4L axis in regulating EGFR protein degradation and LUAD growth, we reasoned that targeting FOXM1 could offer therapeutic advantages in the treatment of EGFR-driven LUAD. To validate this hypothesis preclinically, we sought to identify inhibitors of FOXM1. Firstly, a virtual screening of an FDA-approved Drug Library was used to evaluate the small molecule drugs with the potential to inhibit FOXM1. We subsequently selected five candidates (CAS#20830–75-5, CAS#34580–13-7, CAS#129497–78-5, CAS#29094–61-9 and CAS#182431–12-5) with relatively high scores for further experiment validation (Fig. 7A; Supplementary Fig. S4A). Excitingly, the subsequent western blot analyses revealed that verteporfin (VP, CAS#129497–78-5), a photosensitizer used for photodynamic therapy, was potent to inhibit FOXM1 expression (Supplementary Fig. S4B). In addition, the docking energy of VP-FOXM1 complex (-6.9 kcal/mol) was comparable to that of FDI-6-FOXM1 complex (-6.9 kcal/mol) and RCM-1-FOXM1 complex (-6.6 kcal/mol) (Fig. 7B; Supplementary Fig. S4C, D), which are well-established FOXM1 inhibitors used in scientific researches, thereby partially corroborating our discovery.

We next conducted a rigorous verification to ascertain whether verteporfin-induced FOXM1 inhibition modulates NEDD4L-mediated EGFR proteasomal degradation. Our results showed that verteporfin significantly downregulated FOXM1, upregulated NEDD4L and suppressed the expression of not only wild-type EGFR but also osimertinib-sensitive/resistant EGFR mutant proteins (including EGFR^L858R/T790M^, EGFR^L858R/T790M/C797S^) in a dose dependent manner in H1299, H1975 and H1975OR cells, respectively (Supplementary Fig. 4E). Notably, such verteporfin-induced effects could be significantly reversed by FOXM1 overexpression (Fig. 7C, D). Moreover, verteporfin significantly promoted EGFR protein proteasomal degradation (Fig. 7E; Supplementary Fig. S4F), indicating that verteporfin targets FOXM1 to facilitate NEDD4L-mediated EGFR protein proteasomal degradation.

Further, we explore the functional impact of verteporfin on FOXM1/NEDD4L-modulated EGFR-driven LUAD growth. The in vitro growth assays showed that verteporfin potently suppressed the proliferation of H1299, H1975 and H1975OR cells (Fig. 7F). Consistently, in vivo assays confirmed that verteporfin significantly inhibited tumor growth in H1975OR cell-derived xenografts (Fig. 7G-J). Of note, FOXM1 overexpression or NEDD4L knockdown significantly abrogated the verteporfin-induced inhibition of tumor growth (Fig. 7G-J). Western blot analysis showed that verteporfin inhibited FOXM1 expression, resulting in the upregulation of NEDD4L and downregulation of EGFR and phosphorylated EGFR, which were abrogated by FOXM1 overexpression or NEDD4L knockdown (Fig. 7K).

Taken together, these results suggest that the inhibitory effects of verteporfin on EGFR-driven LUAD growth are at least partially dependent on targeting FOXM1/NEDD4L-mediated EGFR protein proteasomal degradation.

### Combination of verteporfin and osimertinib exerts an additively inhibitory effect on EGFR-mutated LUAD growth compared to monotherapy, both in post-osimertinib resistance and upfront treatment settings

**Fig. 8:**
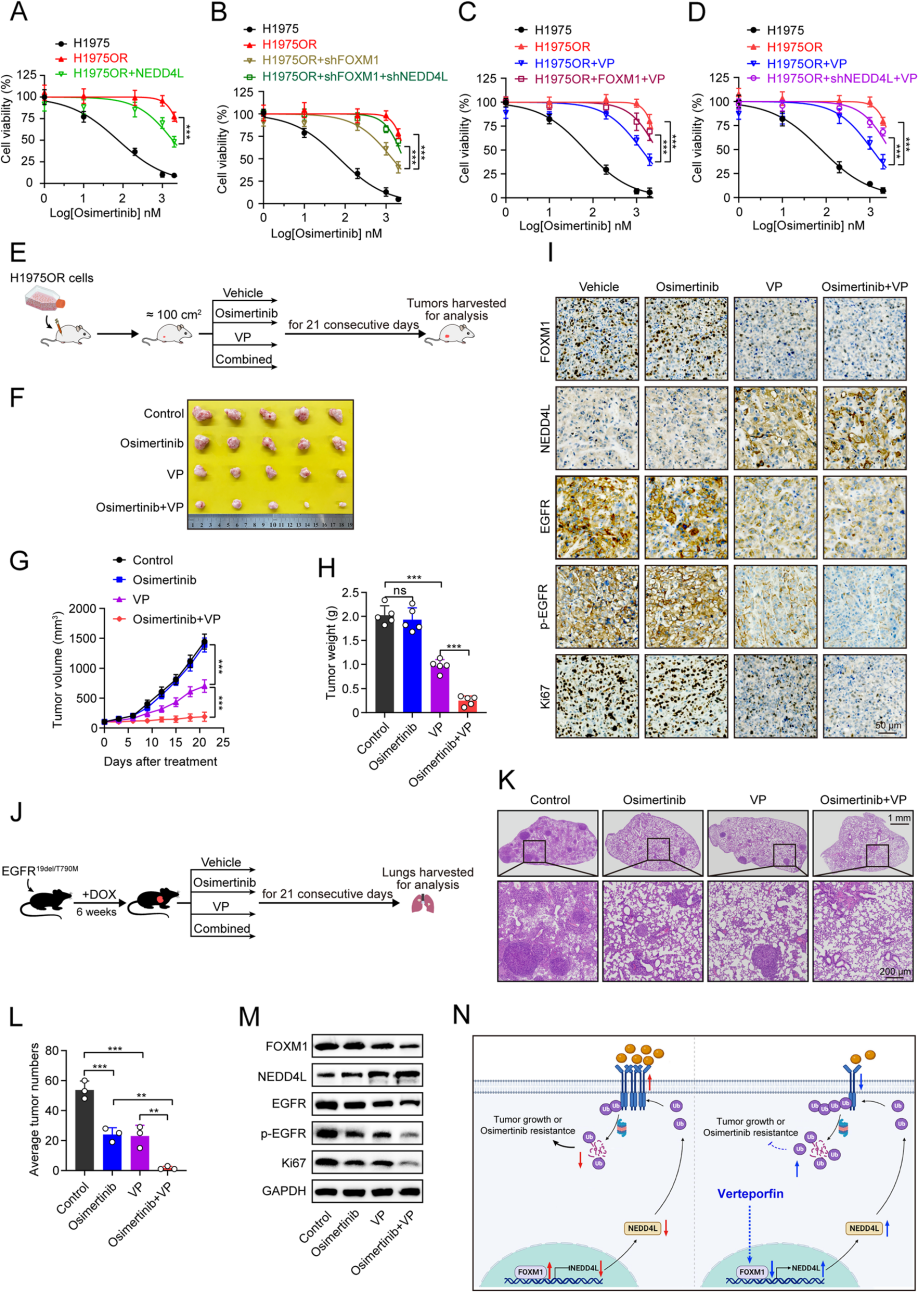
Verteporfin plus osimertinib exert an additively inhibitory effect on EGFR-mutated LUAD growth compared to monotherapy. **A** H1975 or H1975OR cells infected with vector or His-NEDD4L lentivirus were treated with indicated dose of osimertinib for 48 h, followed by CCK8 assays. **B** H1975 or H1975OR cells stably expressing shFOXM1 alone or together with shNEDD4L were treated with an indicated dose of osimertinib for 48 h, followed by CCK8 assays. **C**-**D** H1975 or H1975OR cells incubated with verteporfin (6 µM), or together with FOXM1(**C**) or shNEDD4L(**D**) lentivirus were treated with indicated dose of osimertinib for 48 h, followed by CCK8 assays. **E** Experimental design of CDX model. H1975OR cells were injected subcutaneously in female BALB/c nude mice (*n* = 5/group) for inoculation. When the tumor size reach about 100 mm^3^, mice were treated with osimertinib (5 mg/kg, p.o., daily), or verteporfin (30 mg/kg, i.p., daily), or their combination for a period of 21 days, then the tumors were harvested for analysis. **F** Photos of final shaped tumors. **G** Tumor growth curve. **H** Final tumor weight. **I** IHC analyses of FOXM1, NEDD4L, EGFR, phosphorylated EGFR and Ki67 protein level in final tumor tissues. Scale bar = 10 µm. **J** Experimental design of transgenic mice model. DOX-induced female EGFR^19del/T790M^ transgenic mice (*n* = 3/group) were intraperitoneally treated with osimertinib (2 mg/kg, p.o., daily), or verteporfin (20 mg/kg, i.p., daily), or their combination for a period of 21 days, then the lung samples were harvested for analysis. **K** H&E staining of final lung nodule samples. **L** Numbers of observable nodules in the final lung surface. **M** Western blot analyses of FOXM1, NEDD4L, EGFR, phosphorylated EGFR and Ki67 protein expression in final lung nodule tissues. **N** The proposed working model. VP, verteporfin. ^**^P < 0.01, ^***^P < 0.001, ns, not significant.

Our prior findings showed that verteporfin potently targets FOXM1/NEDD4L to inhibit the protein expression of common EGFR oncogenic mutations, which comprise C797S mutant that confers resistance to osimertinib. As known that osimertinib resistance is a major hurdle for EGFR-mutated LUAD treatment, and there are currently no standard therapeutics approved for osimertinib-resistant LUAD, we thus sought to further investigate the impact of verteporfin-induced FOXM1/NEDD4L/EGFR axis suppression on the response of EGFR^L858R/T790M/C797S^-mediated resistant H1975OR cells to osimertinib in vitro and in vivo. As seen in Fig. 8A-B, the CCK8 results showed that either NEDD4L overexpression or FOXM1 knockdown significantly increased the sensitivity of H1975OR cells to osimertinib, whereas NEDD4L knockdown markedly abrogated the increased sensitivity of H1975OR cells to osimertinib induced by FOXM1 knockdown. Moreover, our subsequent results showed that verteporfin significantly increased the sensitivity of H1975OR cells to osimertinib (Fig. 8C-D). However, either FOXM1 overexpression or NEDD4L knockdown significantly abrogated the verteporfin-induced increased sensitivity of H1975OR cells to osimertinib (Fig. 8C-D). In the H1975OR cell-derived xenograft models, osimertinib alone showed limited inhibitory effect on tumor growth, verteporfin alone had perceptible inhibitory effect on tumor growth, whereas the combination therapy exerted dramatically inhibitory effect (Fig. 8E-H). Subsequent IHC staining results showed that verteporfin monotherapy significantly reduced protein expression of FOXM1, EGFR, p-EGFR, Ki67, and increased protein expression of NEDD4L compared to osimertinib monotherapy (Fig. 8I). Of important note, more obvious inhibitory effects of p-EGFR and Ki67 expression were seen in the combination therapy group compared to monotherapy group (Fig. 8I).

In order to gain insight into whether the combination treatment of verteporfin and osimertinib also confers valuable treatment benefit in osimertinib-sensitive LUAD, we established a doxycycline-inducible EGFR^19del/T790M^ transgenic mice model (Fig. 8J). The in vivo growth assay and western blot showed that the combination of verteporfin and osimertinib exerts an additively inhibitory effect on tumor growth and the expression of p-EGFR and Ki67 compared to either single-agent (Fig. 8K-M). Noteworthily, throughout the treatment period, neither apparent loss of appetite, nausea, vomiting nor significant body weight changes were observed in both nude and transgenic mice (Supplementary Fig. S4G, H).

Taken together, these results suggest that combination of verteporfin and osimertinib exerts an additively inhibitory effect on EGFR-mutated LUAD growth compared to monotherapy, both in post-TKI resistance and upfront treatment settings.

## Discussion

EGFR-TKIs resistance is a big challenge for the treatment of LUAD patients harboring EGFR driver mutations [51]. The significance of EGFR signals in conferring resistance to EGFR-TKIs, either acquired mutation or amplification [51–55], underscores the hypothesis that modulation of EGFR stability represents a novel and alternative treatment strategy. Notably, there is accumulating evidence that the stability, trafficking, and activation of EGFR are intricately influenced by various post-translational modifications, such as phosphorylation, methylation, ubiquitylation and O-GlcNAcylation [56]. Among these modifications, ubiquitination induced by different E3 ubiquitin ligases promotes the degradation of EGFR, thereby suppressing its downstream signals [15, 57]. As an E3 ubiquitin ligase, NEDD4L is known to play a crucial and extensive role in regulating tumor cell functions by ubiquitinating substrate proteins, such as Unc-51-like kinase 1 (ULK1) protein in pancreatic and kidney cancer cells [58, 59] and Notch2 in LUAD [60], thereby facilitating their internalization and turnover [25]. Here, we novelly identified and confirmed that NEDD4L can target not only wild-type EGFR but also osimertinib-sensitive/resistant EGFR mutants for proteasomal degradation, effectively inhibiting EGFR-driven LUAD growth and overcoming osimertinib resistance. The preclinical evidence in this study also demonstrates the important role of FOXM1 in NEDD4L-mediated EGFR protein proteasomal degradation, providing a novel dimension to the complex regulatory network governing EGFR function. While our findings provide substantial evidence that NEDD4L targets EGFR for proteasomal degradation, emerging studies suggest NEDD4L may regulate additional signaling pathways involving in osimertinib resistance. Fox example, it is reported that β-catenin [61] is degraded by NEDD4L through ubiquitination [62, 63] and participates in the process of EGFR-TKI resistance in EGFR mutant lung cancer growth [64]. Notably, osimertinib treatment is known to trigger compensatory upregulation of EGFR family members, including EGFR/ErbB1, HER2/ErbB2, HER3/ErbB3, HER4/ErbB4, driving therapeutic resistance [65, 66]. This raises an important question that whether NEDD4L can modulate the stability of other EGFR family proteins beyond EGFR itself. Interestingly, HER3 has been reported to undergo NEDD4L-mediated proteasomal degradation in breast cancer [67]. Given the established role of HER3 in osimertinib resistance, it would be highly compelling to investigate whether NEDD4L also governs HER3-dependent resistance mechanisms in lung cancer. Although no clear evidence exists regarding the impact of the FOXM1/NEDD4L axis on HER2 or HER4, further investigation is warranted. Besides, the regulation of EGFR protein homeostasis is sophisticated and should not be dependent on NEDD4L alone. E2 ubiquitin-conjugating enzymes, other specific E3 ubiquitin ligases, deubiquitinating enzymes, proteases, as well as components of the proteasomes and lysosomes can be involved [13, 42, 68–70]. Additional modifiers that influence the expression and activities of these enzymes also play a pivotal role [13, 42, 68–70]. It would be of great interest to explore the potential involvement and mechanisms of other factors in regulating EGFR stability in LUAD.

In our current study, FOXM1 was identified as the upstream transcriptional factor of NEDD4L, which belongs to the Winged Helix or Forkhead Box (Fox) family and regulates diverse processes associated with tumorigenesis and development including cell cycle progression, metastasis, and drug resistance [71, 72]. Recent studies, including ours, have highlighted the crucial role of FOXM1 in drug resistance, as evidenced by the aberrant upregulation or variant of FOXM1 mediating TKI resistance in NSCLC [33, 35]. Notably, for the first time, we found that FOXM1 directly binds to the promoter region of NEDD4L and represses its expression, which resulted in an elevation of EGFR protein in LUAD. The demonstration of FOXM1/NEDD4L/EGFR axis provides promising targets for anticancer therapies.

Notably, increasing studies indicate that the specific inhibition of FOXM1 holds promise as an efficient therapeutic strategy for cancer treatment, such as the usages of FOXM1 inhibitors in BRCA-proficient triple-negative breast cancer [73] or NSCLC [26, 74] and specific antibody targeting FOXO3a/FOXM1 axis [33]. However, such FOXM1 inhibitors were primarily research-only drugs. No clinically approved FOXM1 inhibitors are currently available. Excitingly, we have identified verteporfin, an FDA-approved drug for neovascular macular degeneration [75], as a novel inhibitor of FOXM1 with promising potential for clinical application. Verteporfin has been previously proven to inhibit the interactions between YAP/TAZ and the TEA domain transcriptional factor (TEAD), thereby suppressing YAP function and mitigating YAP-induced oncogenic growth [76]. Recently, the anticancer activity of verteporfin has been reported in various cancers, such as ovarian [77], colon [78], pancreatic [79] and thyroid [80] cancers. However, reports on the effects and the underlying anti-cancer mechanisms of verteporfin in LUAD, particularly in the context of EGFR mutation, are sparse and remain to be elucidated. In our study, we found that verteporfin significantly suppresses EGFR-driven LUAD growth. Mechanically, verteporfin facilitates NEDD4L-mediated EGFR protein proteasomal degradation by inhibiting FOXM1 expression, regardless of wild-type EGFR or osimertinib-sensitive/resistant EGFR mutant proteins. Notably, the combination of verteporfin and osimertinib exerts an additively inhibitory effect on EGFR-mutated LUAD growth compared to monotherapy, both in post-TKI resistance and upfront treatment settings. Although the mechanisms underlying the inhibitory effects of verteporfin on FOXM1 expression remain to be fully elucidated, our results with computational prediction as shown in Fig. 7B suggested verteporfin may directly interact with DNA-binding domain [81] of FOXM1, potentially interfering with its transcriptional activity. This proposed mechanism is analogous to that of FDI-6 [82], a known small-molecule inhibitor of FOXM1 that selectively targets its DNA-binding domain to suppress the transcription of FOXM1 target genes. Additionally, verteporfin is a known YAP inhibitor [76], and the regulative effect of YAP on FOXM1 has been reported [83]. While our data suggest a direct effect of verteporfin on FOXM1, we cannot exclude indirect modulation via YAP-dependent pathways. Therefore, it would be of interests to explore whether verteporfin also inhibits the FOXM1/NEDD4L/EGFR axis through targeting YAP in LUAD.

## Conclusions

In conclusion, our study has identified the E3 ubiquitin ligase NEDD4L that targets both wild-type EGFR and osimertinib-sensitive/resistant EGFR mutants for proteasomal degradation, thereby effectively inhibiting EGFR-driven LUAD growth. We found FOXM1 as a critical upstream transcription factor that binds to the promoter of NEDD4L and represses its expression, further promoting tumor growth and osimertinib resistance in LUAD by increasing EGFR protein level. High FOXM1 expression correlates with low NEDD4L expression in LUAD patients, which is associated with poor clinical outcomes. Notably, we further identified that verteporfin, an FDA-approved small molecule drug, as a FOXM1 inhibitor. Verteporfin suppresses FOXM1 to upregulate NEDD4L expression and facilitate EGFR proteasomal degradation, thereby inhibiting EGFR-driven LUAD growth and overcoming osimertinib resistance. Remarkably, the combination of verteporfin and osimertinib shows an additively inhibitory effect on EGFR-mutated LUAD growth compared to monotherapy, both in post-TKI resistance and upfront treatment setting. Taken together, this study demonstrates that FOXM1/NEDD4L axis impaired EGFR proteasomal degradation, and thus contributing to EGFR-driven LUAD growth and osimertinib resistance. Combination therapy incorporating NEDD4L activation may represent a new valued therapeutic strategy for EGFR-driven LUAD and osimertinib resistance (Fig. 8N).

## Supplementary Information

Supplementary Material 1.

## Data Availability

The datasets presented in this study can be found in public online repositories. All other data associated with this study are present in the paper or the supplementary material.

## Abbreviations

LUAD: Lung adenocarcinoma
EGFR: Epidermal growth factor receptor
TKI: Tyrosine kinase inhibitor
NEDD4L: Neural precursor cell-expressed developmentally down-regulated 4 like
FOXM1: Forkhead box protein M1
VP: Verteporfin
OS: Overall survival
DEGs: Different expressed genes
CDX: Cell line-derived xenograft
TMA: Tissue microarrays
PWM: Position weight matrix
IHC: Immunohistochemical
ChIP: Chromatin immunoprecipitation
cDNA: Complementary DNA
IP: Immunoprecipitation
IF: Immunofluorescence
H&E: Hematoxylin and Eosin
TCGA: The Cancer Genome Atlas
GEO: Gene Expression Omnibus
EdU: 5-Ethynyl-2’-deoxyuridine
ICD: Intracellular domain
ECD: Extracellular domain
