# Supplementary material for: Activating NEDD4L suppresses EGFR-driven lung adenocarcinoma growth via facilitating EGFR proteasomal degradation
Source: J Exp Clin Cancer Res. 2025 Oct 21;44:294. doi: 10.1186/s13046-025-03528-y (PMC12541971; doi:10.1186/s13046-025-03528-y)
Supplement: Supplementary file 1 — Supplementary Material 1. [file 13046_2025_3528_MOESM1_ESM.pdf]

Supplementary Fig. S1

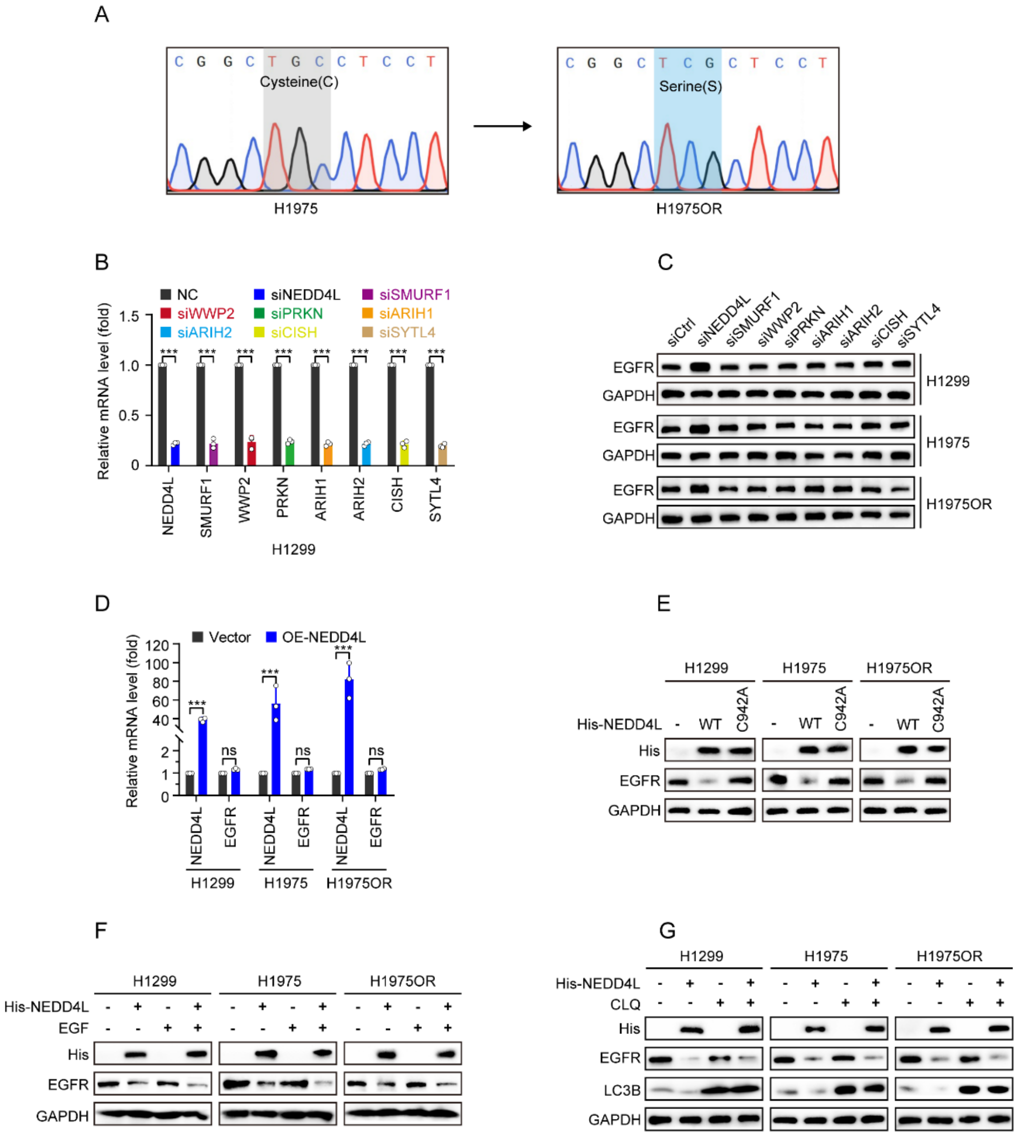

Supplementary Fig. S1 NEDD4L promotes EGFR protein ubiquitination and proteasome-mediated degradation. Related to Fig. 1. (A) Parental cell line (H1975) and the C797S developmental-osimertinib resistant cell line (H1975OR) was validated by sanger sequencing. (B) H1299 cells were respectively transfected with specific siRNAs of NEDD4L, SMURF1, WWP2, PRKN, ARIH1, ARIH2, CISH and SYTL4 for 36 h, followed by qPCR analyses. (C) H1299 cells were respectively transfected with specific siRNAs of NEDD4L, SMURF1, WWP2, PRKN, ARIH1, ARIH2, CISH and SYTL4 for 36 h, followed by western blot analyses. (D) H1299, H1975, or H1975OR cells stably expressing empty vector or OE-NEDD4L were incubated for 24 h, followed by qPCR analyses. (E) H1299, H1975, or H1975OR cells were transfected with His-NEDD4L-wt or C942A mutation expressing plasmids for 36 h, followed by western blot analyses. (F) H1299,

H1975, or H1975OR cells stably expressing empty vector or His-NEDD4L were incubated in serum-free medium for 12 h, and then treated with or without EGF (100 ng/mL) for 10 min, followed by western blot analyses. (G) H1299, H1975, or H1975OR cells stably expressing empty vector or His-NEDD4L were treated with or without chloroquine (CLQ) (50 μM) for 24 h, followed by western blot analyses. $^{***}P < 0.001$, ns, not significant.

Supplementary Fig. S2

A

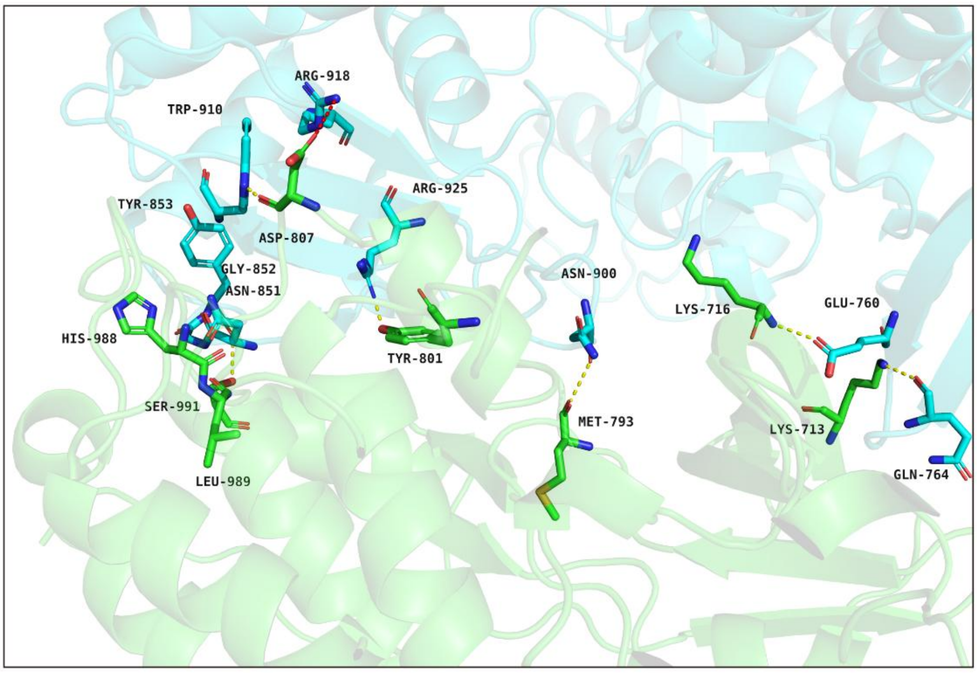

B

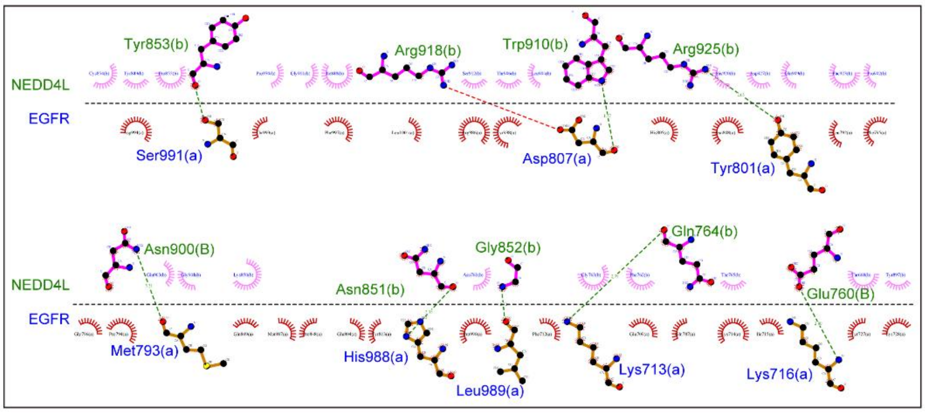

Supplementary Fig. S2 NEDD4L interacts with EGFR. Related to Fig. 2. (A-B) The protein-protein interaction between NEDD4L and EGFR were predicted by ZDOCK 3.0.2. Blue represents NEDD4L, and green represents EGFR. 3D picture (A), 2D picture (B).

Supplementary Fig. S3

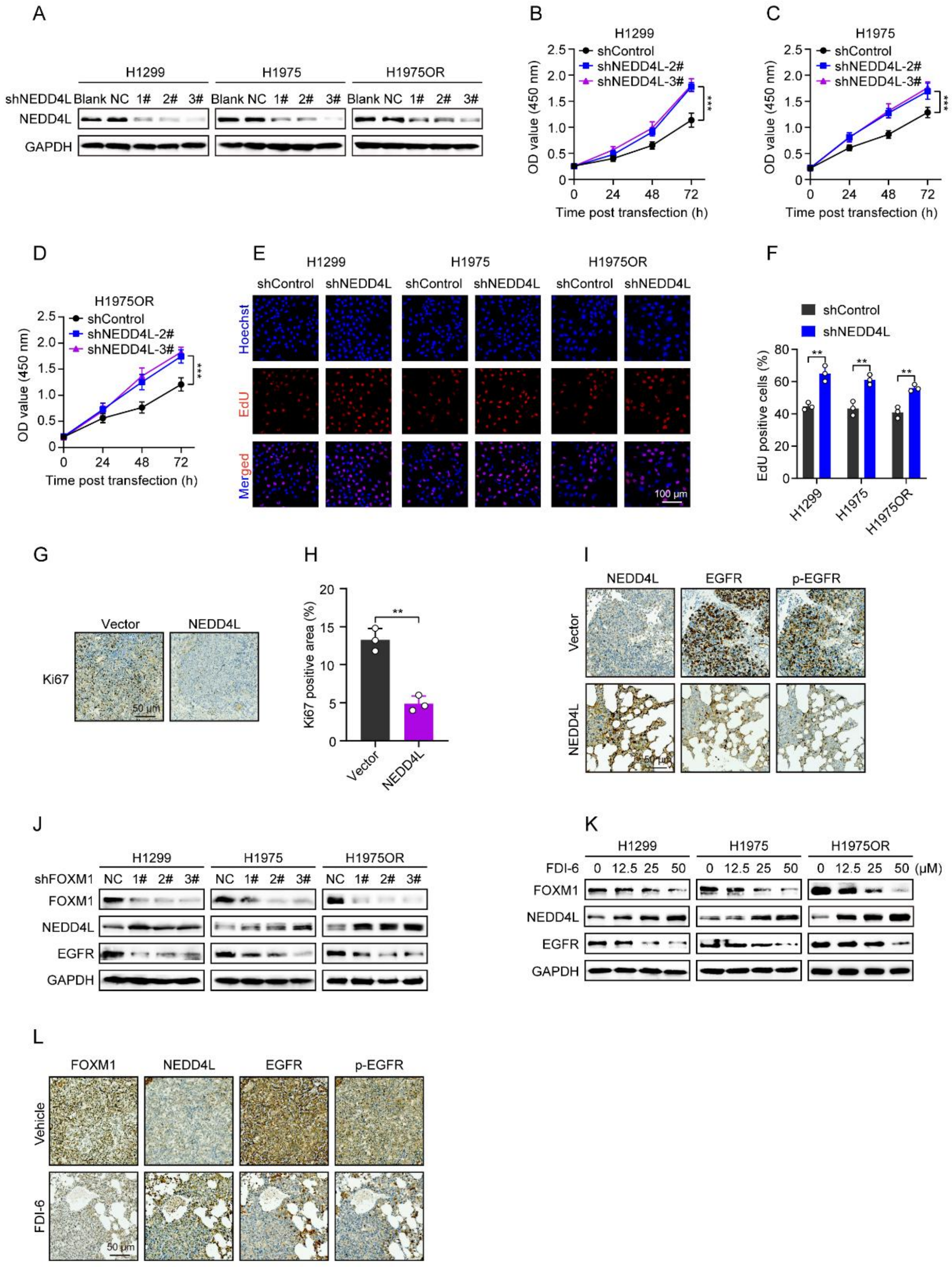

Supplementary Fig.S3 FOXM1 transcriptionally inhibits NEDD4L to promote LUAD growth through increasing EGFR protein expression. Related to Figs. 3, 4, 5. (A) H1299, H1975, or H1975OR cells stably expressing either shControl or shNEDD4L were incubated for 36 h, followed by western blot analyses. (B-E) H1299, H1975, or H1975OR cells stably expressing either shControl or shNEDD4L were incubated for indicated time periods, followed by CCK8 assays (B,

C, D), or EdU assays (E). (F) Quantitative analysis of (E) results. (G) IHC analyses of Ki67 protein expression in final lung nodule tissues. Scale bar = 50 μm. Experimental procedures were seen in Figure 3K. (H) Quantification of Ki67 stained area of (G) results. (I) IHC analyses of NEDD4L, EGFR and phosphorylated EGFR protein expression in final lung nodule tissues. Scale bar = 50 μm. Experimental procedures were seen in Figure 3K. (J) H1299, H1975, or H1975OR cells stably expressing either shControl or shFOXM1 were incubated for 36 h, followed by western blot analyses. (K) H1299, H1975, or H1975OR cells were treated with indicated doses of FDI-6 for 24 h, followed by western blot analyses. (L) IHC analyses of FOXM1, NEDD4L, EGFR and phosphorylated EGFR protein expression in final lung nodule tissues. Scale bar = 50 μm. Experimental procedures can be seen in Fig. 5H. $^{**}P < 0.01$, $^{***}P < 0.001$.

Supplementary Fig. S4

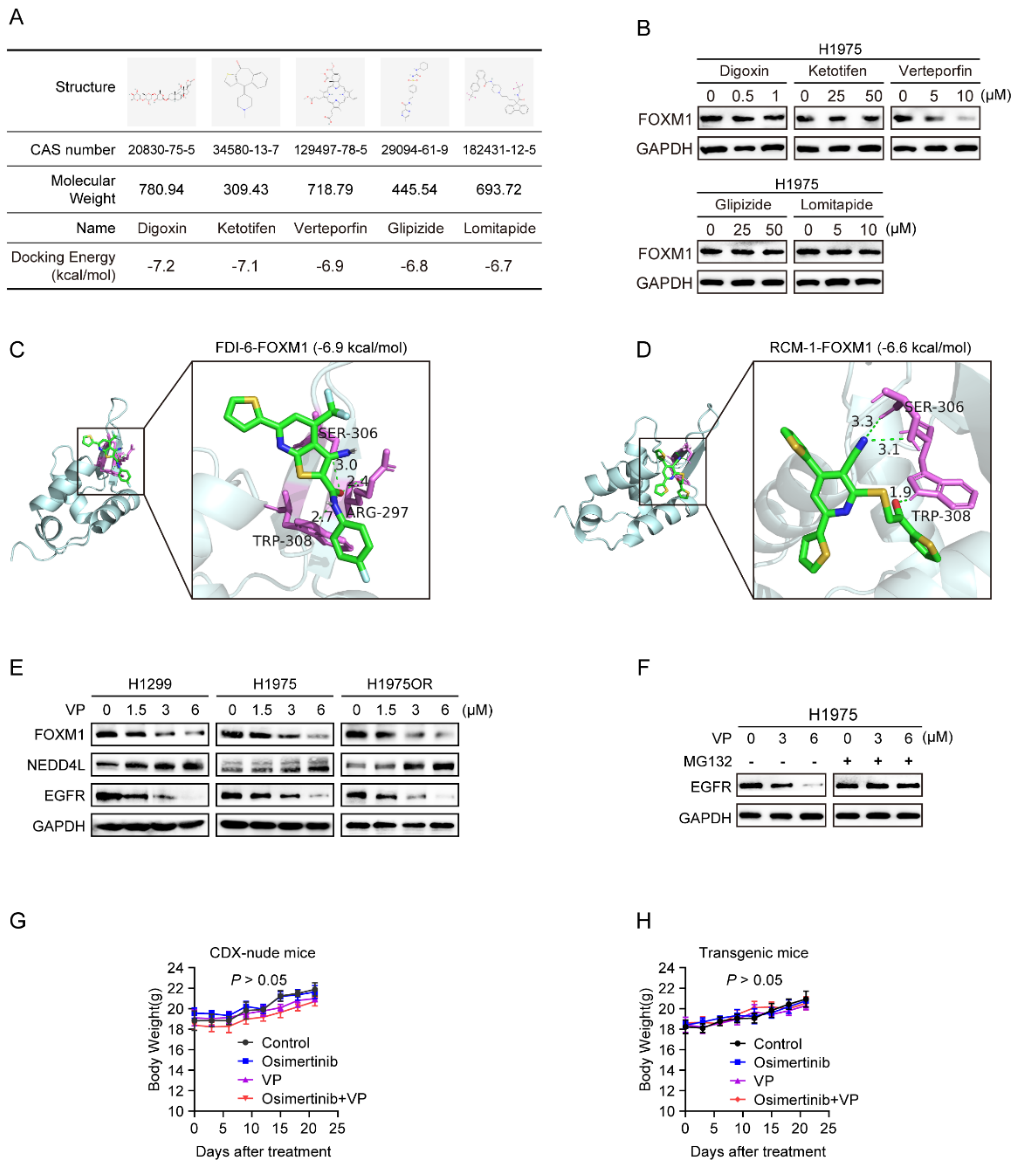

Supplementary Fig. S4 Verteporfin is a novel inhibitor of FOXM1 in facilitating NEDD4L - mediated EGFR proteasomal degradation and inhibiting LUAD growth. Related to Figs. 7, 8. (A) Information of the five FDA-approved candidate small molecule drugs obtained through virtual screening. (B) H1975 cells were treated with indicated doses of the five candidate small molecule drugs for 24 h, respectively, then followed by western blot analyses. (C-D) Molecular docking diagram of the interaction between FOXM1 protein and its commercially available inhibitors (FDI-6 (C), RCM-1 (D)) used in scientific researches. Purple represents FOXM1, and green represents FDI-6 (C) or RCM-1 (D). (E) H1299, H1975, or H1975OR cells were treated with indicated doses of verteporfin for 24 h, followed by western blot analyses. (F) H1975 cells were treated with

indicated doses of verteporfin alone or along with MG132 (20 μM) for 24 h, followed by western blot analyses. (G) Boby weight of CDX-nude mice. (H) Boby weight of transgenic mice. VP, Verteporfin.

Supplementary material

Supplementary Table 1. The detailed basic characteristics of enrolled patients with LUAD

**Characteristics**	**With EGFR amplification (n=29)**	**Without EGFR amplification (n=124)**	***P* value**
**Age, Median [IQR], years**	63[51,68]	62[55,68]	0.54
≤60 years	13(44.8%)	66(53.2%)	
>60 years	16(55.2%)	58(46.8%)	
**Sex, No. (%)**			0.95
Female	17(58.6%)	76(61.3%)	
Male	12(41.4%)	48(38.7%)	
**Smoking Status, No. (%)**			0.80
Never	23(79.3%)	93(75.0%)	
Current/ever	6(20.7%)	31(25.0%)	
**TNM Stage, No. (%)**			0.60
Stage IIIB/IIIC	0(0.0%)	6(4.8%)	
Stage IV	29(100.0%)	118(95.2%)	
**Extra thoracic metastasis, No. (%)**			0.055
Yes	26(89.7%)	87(70.2%)	
No	3(10.3%)	37(29.8%)	
**CNS metastasis, No. (%)**			0.07
Yes	19(65.5%)	56(45.2%)	
No	10(34.5%)	68(54.8%)	
**EGFR activating mutation, No. (%)**			0.37
Exon 19 deletion	15(51.7%)	78(62.9%)	
Exon 21 L858R	14(48.3%)	46(37.1%)	

Abbreviations: CNS metastasis, Central Nervous System (CNS) metastasis.

Supplementary Table 2. Sequences of siRNA/shRNA used in this study (5'-3')

**Target (Human)**	**Usage**	**Sequence (5'to3')**
SMURF1	siRNA	CCGACACTGTGAAAAACAC
WWP2	siRNA	AGGAGGTTCTGCCTGTAATTT
PRKN	siRNA	CAATGATCGGCAGTTTGTTCA
ARIH1	siRNA	CGAGATATTTCCCAAGATT
ARIH2	siRNA	GCTGGATGTGTCTAGGAGATT
CISH	siRNA	GCACAATTATACACTATTTAT
SYTL4	siRNA	GTCTGGTTGTCCATGTGAA
NEDD4L-1#	shRNA	AACCACAACACAAAGTCAC
NEDD4L-2#	siRNA, shRNA	AAGTGGACAATTTAGGCCGAA
NEDD4L-3#	shRNA	CCTCTGTAATGAGGATCATTT
FOXM1-1#	shRNA	GCCCAACAGGAGTCTAATCAA
FOXM1-2#	shRNA	GCCAATCGTTCTCTGACAGAA
FOXM1-3#	shRNA	GCTCATACCTGGTACCTAT
EGFR	shRNA	GGCTGGTTATGTCCTCATT
Control1	siRNA	TTCTCCGAACGTGTCACGT
Control2	shRNA	TTGCAGGGTGGTCCGTGTAAA

Supplementary Table 3. Sequences of qPCR primers used in this study (5'-3')

**Gene (Human)**	**Primer Sequence (5'to3')**
SMURF1-F	CTGTACTGGACCACACCTTCTG
SMURF1-R	CTAAGAACTGGGCTTCGATTC
WWP2-F	GAGATGGACAACGAGAAG
WWP2-R	CTCCTCAATGGCATACAG
PRKN-F	AAATGCCCAGACAAGATGCC
PRKN-R	GGCCTCTCACGACTGAGTT
ARIH1-F	AATGTATCCGGGAGGTCAACG
ARIH1-R	GGCGTGTTCGAGACTTTTTACT
ARIH2-F	TGTATGCAGTTTGTGCGAAAGG
ARIH2-R	GCCATGCAAGAGACTCCCA
CISH-F	CCTCTGCGTTCAGGGGTGAG
CISH-R	TTGGAGCGCAGCAGTCTAAA
SYTL4-F	ATGTGCCTGGAACTGACTGT
SYTL4-R	ACTGATCCCAGTGCCAACAC
NEDD4L-F	GGTTCCAATGGTCCTCAGCT
NEDD4L-R	GCATTTTCCACGGCCATGAG
FOXM1-F	ACCCAAACCAGCTATGATGCC
FOXM1-R	TCCCGTTTCTGCTCGCAAAG
EGFR-F	GGACTCTGGATCCCAGAAGGTG
EGFR-R	GCTGGCCATCACGTAGGCTT
GAPDH-F	GTCTCCTCTGACTTCAACAGCG
GAPDH-R	ACCACCCTGTTGCTGTAGCCAA
NEDD4L_promoter-F	AGGTTCAAGGGATCCTCATG
NEDD4L_promoter-R	GGTGGCTCAAACCTGTAATC
